# DeepSRE: Identification of sterol responsive elements and nuclear transcription factors Y proximity in human DNA by Convolutional Neural Network analysis

**DOI:** 10.1371/journal.pone.0247402

**Published:** 2021-03-04

**Authors:** Davide Noto, Antonina Giammanco, Rossella Spina, Francesca Fayer, Angelo B. Cefalù, Maurizio R. Averna

**Affiliations:** Department of Health Promotion, Mother and Child Care, Internal Medicine and Medical Specialties, University of Palermo, Palermo, Italy; Taipei Medical University, TAIWAN

## Abstract

SREBP1 and 2, are cholesterol sensors able to modulate cholesterol-related gene expression responses. SREBPs binding sites are characterized by the presence of multiple target sequences as SRE, NFY and SP1, that can be arranged differently in different genes, so that it is not easy to identify the binding site on the basis of direct DNA sequence analysis. This paper presents a complete workflow based on a one-dimensional Convolutional Neural Network (CNN) model able to detect putative SREBPs binding sites irrespective of target elements arrangements. The strategy is based on the recognition of SRE linked (less than 250 bp) to NFY sequences according to chromosomal localization derived from TF Immunoprecipitation (TF ChIP) experiments. The CNN is trained with several 100 bp sequences containing both SRE and NF-Y. Once trained, the model is used to predict the presence of SRE-NFY in the first 500 bp of all the known gene promoters. Finally, genes are grouped according to biological process and the processes enriched in genes containing SRE-NFY in their promoters are analyzed in details. This workflow allowed to identify biological processes enriched in SRE containing genes not directly linked to cholesterol metabolism and possible novel DNA patterns able to fill in for missing classical SRE sequences.

## Introduction

Deep Learning techniques have been widely applied to the study of nucleic acids and protein sequences in recent years. The structure of DNA, RNA and proteins, characterized by repetitions of elemental monomers, makes their primary sequences suitable to be converted to numeric matrices and then fed into neural networks. Some studies focused on identification of particular responsive elements in the DNA, such as enhancers [1–3], while in other cases Deep Learning has been used to predict secondary structures from primary sequences [4], or putative interactions between molecules. In particular protein-protein interactions [5], RNA-protein interactions [6], and DNA–protein interactions have been investigated.

Convolutional Neural Networks (CNN) have been widely used for these purposes [1–5] because they are position-invariant, meaning that a single feature (i.e., a particular stretch of elements) can be detected irrespectively of its position within a sequence. This is very relevant in the analysis of transcription factors (TF) binding sites located in gene promoters. TF responsive elements are often composed of small sequence blocks that are in strict proximity, but that can be separated each other by a variable number of bases. Gene promoters present such elements in variable conformations, switched in position in the same strand, or even facing each other in opposite strands. For these reasons CNNs are the most used neural models to analyze non-contiguous structures, even if other models, as the Recurrent Neural Networks (RNNs) have been also used [2]. The CNN have been used as image classificators, due to their ability to identify patterns in images irrespective of their locations. Since images are bi-dimensional matrices of pixels (width x height), then two-dimensional neural models (CNN-2D) have been used in such cases. In the case of nucleic acids, a primary sequence is a linear vector expressed in a single dimension, so a simplification of CNN, called CNN-1D, has been developed.

In the present paper we investigated the structure and occurrence of the sterol responsive element (SRE), a peculiar sterol sensor located in the promoters of many genes involved in cholesterol metabolism, in particular in those involved in cholesterol biosynthesis. SRE is a sequence of bases that binds to SREBP1 and SREBP2, two transcription factors that are released by the “Golgi” when the intracellular cholesterol pool is depleted [7], triggering a coordinated cellular response aimed to restore the optimal intracellular cholesterol pool. The SRE sequence is not fixed, it can change in some bases, as it happens for most of the responsive elements, and it is often flanked by other elements, as the nuclear factor Y (NFY), and by the specificity protein 1 (SP-1), that can be present in different copies. Looking at different promoters, it has been found that SRE surrounding elements can be aggregated in a modular fashion, so that the resulting structure is never the same in different promoters [7]. For this reason, we tested the hypothesis that the CNN-1D, used to identify DNA structures as enhancers or TF binding sites (1–5), might be a useful tool to investigate the SRE-NFY architecture and to detect the presence of complex sterol responsive structures in promoters of genes not directly linked to cholesterol metabolism.

## Methods

### Software

The R programming language (https://cran.r-project.org/) was used to write all the routines of the proposed workflow. R was run under RStudio Version 1.3.959, a dedicated integrated development environment (IDE) for R (https://rstudio.com/). The CNN-1D was built in Rstudio using Keras’ version 2.3.0.0.9000 with Tensorflow 1.13.1 (https://www.tensorflow.org/) as backend (https://keras.rstudio.com/). The data underlying this article (R scripts and text supporting files, help files) are freely available at doi.org/10.17504/protocols.io.bm4fk8tn. A copy of the help file is also available as Supplemental Material, while an html version is available at http://www1.unipa.it/cermmet.sicilia/index.php/it/software/159-deepsre-finding-sre-nfy-sensors-in-promoters-with-deep-learning.

### SRE and NFY chromosomal locations

Chromosomal locations harboring putative SRE and NFY sites were identified by interrogating the comprehensive set of human transcription factor binding sites from Chromatin immune precipitation (ChIP)-seq experiments generated by the ENCODE Consortium and held at the University of California, Santa Clara (UCSC) http://genome.ucsc.edu/. ChIP peaks for SRE and NFY were downloaded using the table browser feature of the UCSC genome browser site. The GRCh38/hg38 genomic coordinate system was used. A set of control ChIP peaks was also collected from the same source. The list of ENCODE TF peaks and their specifications is presented in S1 Table.

A set of sequences of 100 bp spanning from -50 to +50 bp around the chromatin chromosomal peaks were obtained by a custom R routine that extracted the DNA sequence from whole chromosome sequences downloaded in FASTA format from UCSC at https://hgdownload.soe.ucsc.edu/goldenPath/hg38/chromosomes/.

Another R routine selected all the SRE ChIP peaks that presented a corresponding NFY peak in close proximity (less than 250 bp), so that 1301 SRE-NFY couples were selected. The distribution of the SRE–NFY distances in bp is presented in S1 Fig. A set of validation data was obtained by a ChIP-set experiment from a published paper presenting data on SRE, NFY, and SP-1 binding sites colocalization [8]. These data were not used neither to train nor to validate the model, but were used as an independent source to check the presence of SRE or NFY binding sites in promoter sequences.

### Data augmentation by phylogenetic score

The 1301 SRE-NFY couples had to be augmented to match with the 140,343 control sequences. The augmentation process usually uses random permutation of bases to introduce a low level of diversity between the original and the augmented sample. In our setting, augmentation was based on phylogenetic conservation of bases. Phylogenetic scores (phylogenetic p-values) from the PHAST package were downloaded from http://hgdownload.cse.ucsc.edu/goldenpath/hg38/phyloP100way/. A R routine created one thousand of copies of each SRE-NFY sequence introducing 20 mutations in each sequence based on the “roulette wheel selection” algorithm, creating wheel slices proportional the inverse of the phylogenetic scores, so that the most conserved bases had less probability to be mutated when spinning the virtual wheel. At the end of the procedure the SRE-NFY couples accounted to 131,401 (positive) samples, vs. the 140,343 control (negative) samples. The data set was split in training data set and validation data set with a ratio 3:1 by random sampling of the data set. All sequences were shuffled before feeding them to the CNN.

### Data augmentation by reverse strand complementing

The resulting sequences were 100 bp long, but they were doubled in size by including the reverse sequence concatenated with the forward sequence, as suggested by a previous publication [9] showing that the reverse complementation increased the performance of the CNN model. No comparative performance testing was made in our setting to prove if complementation effectively increased model performance.

### Neural network

**Fig 1:**
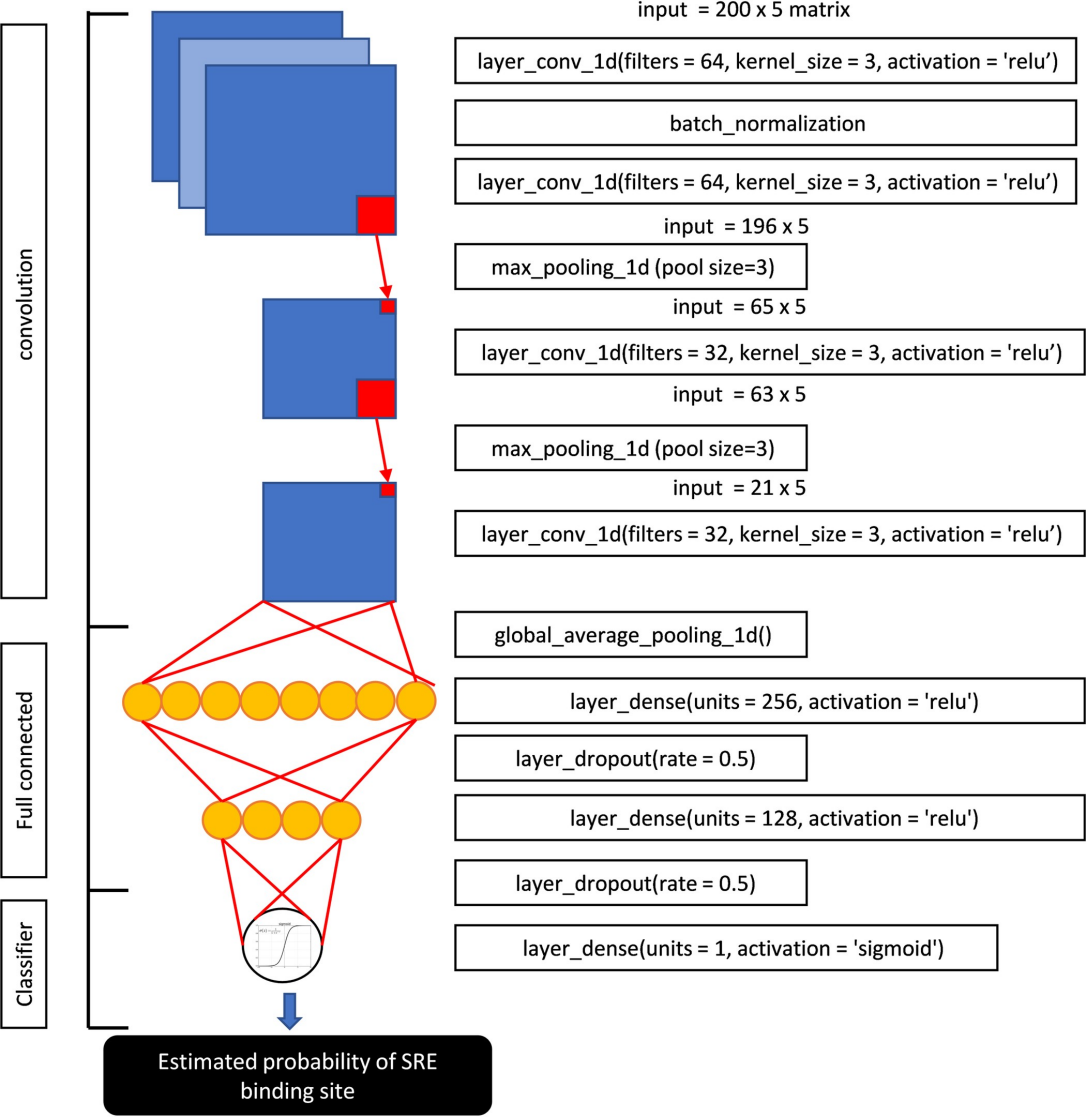
Architecture of the CNN-1D model created by Keras.

The architecture of the CNN model is represented in Fig 1, while the model dimensions are presented in S2 Fig. The primary DNA sequences were converted automatically to two-dimensional matrices by Keras according to the “one-hot encoding” procedure [10], so that every base was represented by a binary sequence of 5 digits (a = “01000”, t =“00100”, c = “00010, g = “00001”, “null” = “10000”). The final dimensions of the sequence matrices were [200x5]. The CNN model was tuned by modulating two model parameters: the number of CNN filters (32,64,128) and the kernel size (3,6,12). As shown in Fig 1, the last layer of Fig 1 is a one-unit dense layer activated by a sigmoid function estimating the probability of a SRE binding site in the DNA sequence.

The model was fit using a custom generator that loaded only batches of 512 sequences that were converted to matrices, hot-encoded and fed to the model on the fly. The model needed 80 iterations divided in three blocks of 40,30,10 iterations respectively to achieve optimal results. For each block the process stopped with an “early stopping callback” procedure that stepped to the next iteration block if the validation accuracy did not increase after 15,10,5 iterations respectively.

### Scanning of human promoters for SRE-NFY sites

Complete sequences of the first 500 bases of the human promoters (n = 28,224) were obtained by extracting sequences of 500 bases ahead the gene transcription starts, using the transcript coordinates contained in the “.bed” files from UCSC Genome Browser (https://hgdownload.soe.ucsc.edu/goldenPath/hg38/chromosomes/.). For genes located in the reverse strand, the R routine considered the 500 bases following the end of transcription and reverse-complemented the bases so that all the promoter sequences were in forward direction. Another R routine scanned each of the 28,224 promoter sequences in blocks of 100 bases that were fed to the CNN 1D model in search of possible SRE-NFY sites. From the base at -500 from the transcription start, to the base at -100, the R routine extracted partially overlapping sequences of 100 bases in steps of 20 bases sliding over the promoter sequence, and evaluated the probability of each sequence according to the CNN. A positive match for a SRE-NFY site was considered when at least three consecutive sequences obtained a mean probability score > 0.9. This arbitrary pattern was determined after manually revising the model prediction of promoters including positive sequences used in the training set.

### Evaluating the enrichment of SRE-NFY sites according to Gene Ontology terms

A list of Gene Ontology (GO) terms linked to human genes was obtained from Quick GO site (https://www.ebi.ac.uk/QuickGO/) by the European Bioinformatics Institute (EMBL-EBI) using only the “Biological Process” annotation. Enrichment in SRE-NFY predictions of each biological process was calculated by counting the number of promoters with positive SRE-NFY estimation divided the total number of genes listed in the GO group, and comparing such ratio with the (number of positive genes / total number of genes) ratio of all the remaining genes not listed in the GO group. The enrichment in predicted SRE-NFY couples was expressed by the odds ratio with 95% confidence interval calculated on the resulting 2x2 table by the “oddsratio.fisher” function of the “epitools” R package.

### Evaluation of the relevant features of CNN 1D model by occlusion of sequences with highest probability of containing a putative SRE-NFY site

The interpretation of the model output was performed by the occlusion technique. The model was fed with the sequences from the promoters with the highest probability of predicted SRE-NFY sites. Again, the 500 bp long promoters were split in chunks of 100 bp each. For every chunk, the model probability was re-calculated one hundred times after “occluding” (i.e., zeroing) a single base at each step. The procedure resulted in a vector of 100 probabilities, one for each base of the 100 bp sequence. The most relevant bases were considered those that caused the highest loss of probability when occluded. As mentioned, the same protocol used for the promoter analysis was used here to increase the sensitivity of the procedure, i.e. the promoters were divided in sequences of 100 bp, in steps of 5 bp, so that each sequence shared the 90% of the bases with the following one, which was forward-shifted by 5 bases. The 100 bp sequences exhibiting the highest mean probabilities and showing the sharpest drop in model probabilities after occlusion were manually revised and averaged to obtain a single plot of mean probabilities, with sharp peaks in correspondence of the bases that determined the highest losses. The sequences surrounding the relevant bases were examined for the presence of transcription factors sites by the JASPAR Genome Tracks tool (http://jaspar.genereg.net/) in conjunction with UCSC Genome browser (http://genome.ucsc.edu/). Publications identifying SRE and NFY sites in cholesterologenic genes by dedicated experiments were considered in some cases [7, 8, 11].

## Results

**Fig 2:**
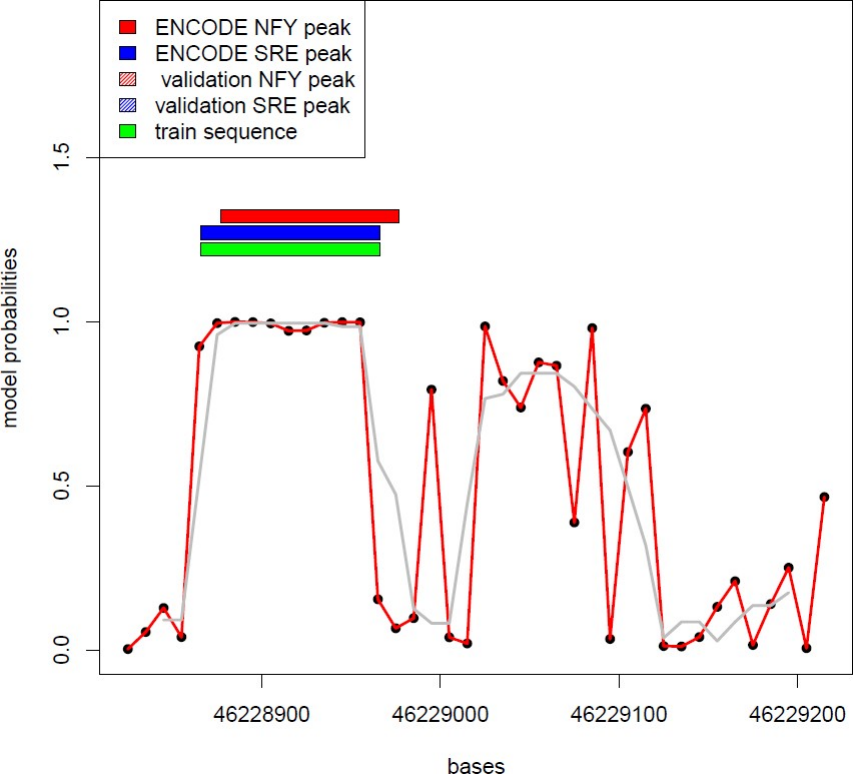
Model probabilities of occurrence of an SRE-NFY couple in the first 500 bases of the Lanosterol Synthase (*LSS*) promoter.

The performance of the model is presented in S2 Table. The model was fed with 1314 positive sequences containing both SRE and NFY TFChip peaks within 250 bp of distance, that were augmented to 131401 as described in Methods, and 136302 negative sequences. The model was stopped by early stopping in less than 45 iterations, and reached accuracy = 0.956, precision = 0.954, recall = 0.957, F1 score = 0.955, at the end of the fitting process. The analysis of 28,224 promoters was performed as described in methods, and it resulted in a typical pattern described in Fig 2 for the Lanosterol Synthase (*LSS*) gene.

Fig 2 shows the model probabilities evaluated for the LSS promoter. The promoter was split in 40 sequences of 100 bases with a partial overlap (each sequence is shifted by 20 bases from the previous one). The x-axis shows the physical chromosomal position in bp. The y-axis shows the probability of the occurrence of an SRE-NFY couple according to the model. Individual model estimations are shown as black dots linked by a red line. The gray line represents the smoothing of the curve. The blue, red and green bars indicate the presence of a TF-Chip SRE binding site, a TF-Chip NFY binding site and the region of the promoter used to train the model, respectively. The shaded bars indicate, if present, the occurrence of SRE or NFY binding sites according to an independent ChIP experiment source [8].

The Fig 2 shows that a positive SRE-NFY site is characterized by consecutive model predictions with p-value > 0.9. In case of LSS we counted 7 points above the 0.9 probability threshold, but the revision of many genes showed that a minimum of 3 consecutive points with p-value > 0.9 was typical of an SRE-NFY couple detected in the promoters used as true positives in the training set.

Consequently, all the promoters were screened and ranked according to this pattern.

To understand if any cluster of genes representative of a biological process was enriched in genes positive for SRE-NFY occurrence, an automated procedure was created to evaluate the odds ratios of all the biological process (indicated by GOterms) to be enriched in genes containing SRE-NFY patterns. The Table 1 shows the thirty biological processes with the highest odds ratios, the relative p-values and the raw numbers used to calculate the odds ratios.

**Table 1:** Biological processes with the highest enrichment in genes containing estimated SRE-NFY couples.

GO code	GO biological process	Odds ratio (CI)	P-value	In-group ratio	Out-group ratio
6298	mismatch repair	6.89 (3.14–14.87)	<0.001	0.82 (14/17)	0.12 (3008/25185)
51252	regulation of RNA metabolic process	5.98 (2.86–12.19)	<0.001	0.71 (15/21)	0.12 (3007/25181)
6695	cholesterol biosynthetic process	5.58 (2.77–10.97)	<0.001	0.67 (16/24)	0.12 (3006/25178)
45737	positive regulation of cyclin-dependent protein serine/threonine kinase activity	5.28 (2.34–11.47)	<0.001	0.63 (12/19)	0.12 (3010/25183)
34620	cellular response to unfolded protein	5.23 (2.12–12.26)	<0.001	0.63 (10/16)	0.12 (3012/25186)
45540	regulation of cholesterol biosynthetic process	5.18 (2.38–10.85)	<0.001	0.62 (13/21)	0.12 (3009/25181)
45292	mRNA cis splicing; via spliceosome	4.64 (1.91–10.62)	<0.001	0.56 (10/18)	0.12 (3012/25184)
51085	chaperone mediated protein folding requiring cofactor	4.6 (1.99–10.08)	<0.001	0.55 (11/20)	0.12 (3011/25182)
97352	autophagosome maturation	4.56 (2.06–9.64)	<0.001	0.55 (12/22)	0.12 (3010/25180)
6310	DNA recombination	4.51 (2.52–7.88)	<0.001	0.54 (21/39)	0.12 (3001/25163)
16070	RNA metabolic process	4.47 (2.27–8.47)	<0.001	0.53 (16/30)	0.12 (3006/25172)
6986	response to unfolded protein	4.44 (2.35–8.09)	<0.001	0.53 (18/34)	0.12 (3004/25168)
6611	protein export from nucleus	4.4 (1.83–9.96)	<0.001	0.53 (10/19)	0.12 (3012/25183)
70	mitotic sister chromatid segregation	4.36 (1.98–9.15)	<0.001	0.52 (12/23)	0.12 (3010/25179)
32008	positive regulation of TOR signaling	3.98 (1.67–8.84)	0.001038	0.48 (10/21)	0.12 (3012/25181)
15867	ATP transport	3.96 (1.58–9.19)	0.001892	0.47 (9/19)	0.12 (3013/25183)
32007	negative regulation of TOR signaling	3.96 (1.58–9.19)	0.001892	0.47 (9/19)	0.12 (3013/25183)
48538	thymus development	3.9 (1.91–7.6)	<0.001	0.47 (14/30)	0.12 (3008/25172)
45	autophagosome assembly	3.87 (2.08–6.93)	<0.001	0.46 (18/39)	0.12 (3004/25163)
7017	microtubule-based process	3.8 (1.6–8.37)	0.001366	0.45 (10/22)	0.12 (3012/25180)
34080	CENP-A containing nucleosome assembly	3.76 (1.51–8.65)	0.002486	0.45 (9/20)	0.12 (3013/25182)
7062	sister chromatid cohesion	3.71 (1.4–8.99)	0.004544	0.44 (8/18)	0.12 (3014/25184)
18345	protein palmitoylation	3.71 (1.4–8.99)	0.004544	0.44 (8/18)	0.12 (3014/25184)
48705	skeletal system morphogenesis	3.63 (1.54–7.95)	0.001774	0.43 (10/23)	0.12 (3012/25179)
30968	endoplasmic reticulum unfolded protein response	3.59 (1.82–6.75)	<0.001	0.43 (15/35)	0.12 (3007/25167)
6342	chromatin silencing	3.58 (1.44–8.17)	0.003219	0.43 (9/21)	0.12 (3013/25181)
6626	protein targeting to mitochondrion	3.58 (1.44–8.17)	0.003219	0.43 (9/21)	0.12 (3013/25181)
31047	gene silencing by RNA	3.58 (1.44–8.17)	0.003219	0.43 (9/21)	0.12 (3013/25181)
8654	phospholipid biosynthetic process	3.52 (1.33–8.42)	0.005861	0.42 (8/19)	0.12 (3014/25183)
48255	mRNA stabilization	3.52 (1.33–8.42)	0.005861	0.42 (8/19)	0.12 (3014/25183)

The table presents the enrichment of biological processes (coded by Gene Ontology) in genes containing the SRE-NFY pattern detected by CNN model. GO code = Gene Ontology numeric code of the GO process, OR = odds ratio with CI = confidence intervals in brackets. p-value = odds ratio Fisher test p-value, in-group ratio = ratio in the GO biological process group of: number of genes with SRE-NFY detected / number of genes negative for SRE-NFY detection. Out-group ratio = ratio in all the remaining biological processes used as control group of: number of genes with SRE-NFY detected / number of genes negative for SRE-NFY (absolute number of genes in brackets).

**Fig 3:**
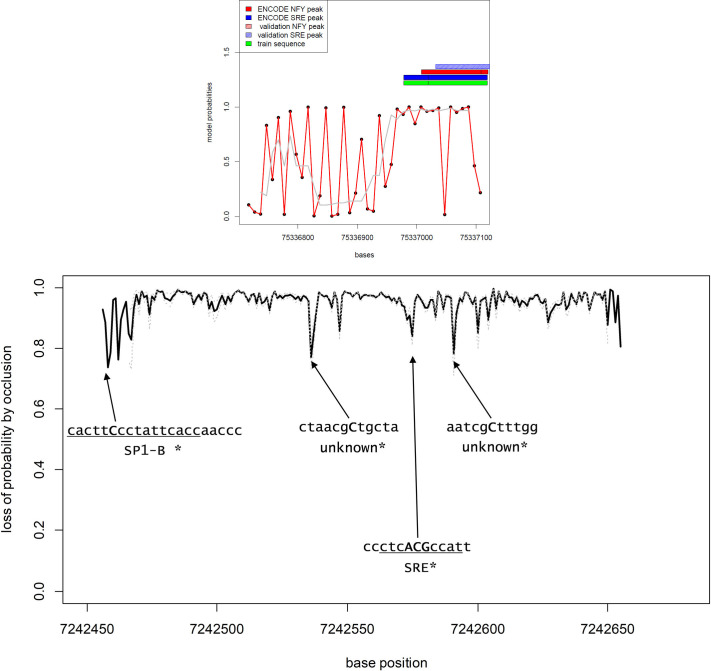
Evaluation of the HMGCR promoter by the CNN 1-D model.

To understand which sequences activated the CNN model, the promoter sequences in which SRE-NFY sites were predicted by the model were analyzed by the occlusion procedure outlined in the method section. Fig 3 shows the example of the hydroxy-methyl-glutaryl-CoA-reductase (*HMGCR*) promoter, in which both SRE and NFY binding sites were detected by the ENCODE TF-ChIP (Panel A, blue and red bars), and that was used as positive sequence to train the model (Panel A, green bar). More, also the independent ChIP experiment of reference [8] found a corresponding SRE site (shaded blue bar).

Panel A: Model probabilities of occurrence of an SRE-NFY couple in the first 500 bases of the HMGCR promoter. The promoter was split in 40 sequences of 100 bases with a partial overlap (each sequence is shifted by 20 bases from the previous one. The x-axis shows the physical chromosomal position in bp. The y-axis shows the probability of the occurrence of an SRE-NFY couple according to the model. Individual model estimations are shown as black dots linked by a red line. The gray line represents the smoothing of the curve. The blue, red and green bars indicate the presence of a TF-Chip SRE binding site, a TF-Chip NFY binding site and the region of the promoter used to train the model, respectively. The shaded bars indicate, if present, the occurrence of SRE or NFY binding sites according to an independent ChIP experiment source [8].

Panel B: Loss of model probability by averaged single base occlusion. The panel shows the loss of probability by occluding iteratively each base of 100 bp chunks from the 500 bp promoter. Multiple sequences of 100 bases partially overlapping (separated by 5 bp) were analyzed and the best results were averaged to produce the plot (see methods). The sequences corresponding to the highest losses (negative peaks) were screened in search of relevant binding sites. The capital letters in bold represent the bases corresponding to the peaks, while the underlined sequence indicate the TF binding site identified by reference [11].

The occlusion procedure applied to the *HMGCR* promoter showed that the model was activated by short sequences that were effectively part of SRE of NFY binding sites.

**Fig 4:**
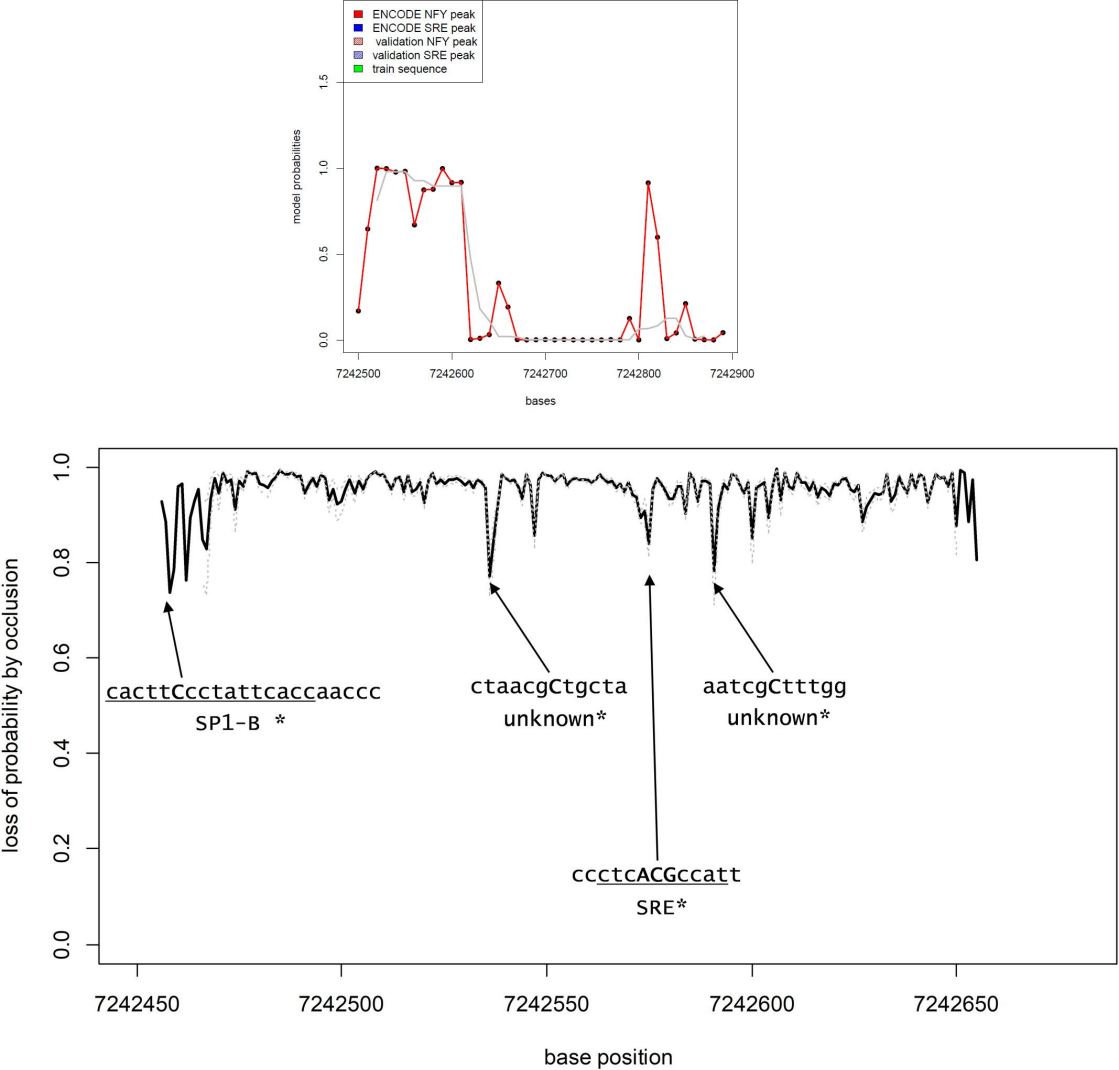
Evaluation of the *GABARAP* promoter by the CNN 1-D model.

Fig 4 shows the example of the *GABARAP* promoter, in which no SRE or NFY sites were found in TF-ChIP experiment, and that did not contain sequences included in the training set.

Panel A: Model probabilities of occurrence of an SRE-NFY couple in the first 500 bases of the GABARAP promoter. The promoter was split in 40 sequences of 100 bases with a partial overlap (each sequence is shifted by 20 bases from the previous one. The x-axis shows the physical chromosomal position in bp. The y-axis shows the probability of the occurrence of an SRE-NFY couple according to the model. Individual model estimations are shown as black dots linked by a red line. The gray line represents the smoothing of the curve. The blue, red and green bars (absent in this promoter) indicate the presence of a TF-Chip SRE binding site, a TF-Chip NFY binding site and the region of the promoter used to train the model, respectively. The shaded bars indicate, if present, the occurrence of SRE or NFY binding sites according to an independent ChIP experiment source [8].

Panel B: Loss of model probability by averaged single base occlusion. The panel shows the loss of probability by occluding iteratively each base of 100 bp chunks from the 500 bp promoter. Multiple sequences of 100 bases partially overlapping (separated by 5 bp) were analyzed and the best results were averaged to produce the plot (see methods). The sequences corresponding to the highest losses (negative peaks) were screened in search of relevant binding sites. The capital letters in bold represent the bases corresponding to the peaks, while the underlined sequence indicate the TF binding site identified by the JASPAR database*.

The Fig 4 shows that the model predicted a high probability of an SRE-NFY couple in absence of SRE or NFY peaks detected by TF-ChIP analysis. Still, a putative SRE binding site was present in the sequence according to JASPAR database and it was considered a relevant feature by the CNN-1D model. A NFY site was not found, but a SP-1 site seemed to activate the CNN model. Other promoters of genes from the most relevant GO groups are presented in S3–S11 Figs. Transcription factors binding sites graphical representations according to JASPAR are presented in S12 Fig.

## Discussion

Convolutional Neural Networks have been used in Genetics to detect particular nucleic acids sequences, using the property of CNNs to discover non-contiguous patterns in sequences, as it happens for enhancers [1–3, 12], or long non-coding RNA [13]. The strategy to convert the genomic sequence into a “hot-encoded”, [5 digits (n = 0, a = 1, c = 2, g = 3, t = 4) x sequence length], two-dimensional matrix has made the genetic sequence interpretable by CNN, that was originally developed to analyze image pixels in a three-dimensional prospective. CNNs are basically very powerful classifiers, but the rules used by the neural networks to accomplish their tasks are not always meaningful to humans. Then, it is important to train the model with an appropriate training set to obtain a predictive model able to recognize the features that we consider biologically relevant. The present paper was aimed to find novel gene promoters responsive to cholesterol signaling “via” the SREBP/SCAP/SRE cell machinery [8]. The analysis of the SRE binding sites has been performed in the past by ChIP experiments, and it was observed that most of the cholesterol responsive promoters presented the co-occurrence of SRE, NFY and SP1 sites in close proximity [8, 11]. Starting from these premises, a CNN model was designed to be trained with short promoter sequences in which a SRE putative site, identified as a SRE ChIP peak, was located in close proximity of a NFY site (proximity was set arbitrarily at less than 250 bp). This procedure identified 1301 SRE-NFY couples from the ENCODE dataset, and the number of samples was augmented using a novel strategy that permuted iteratively a small number of bases not randomly, but according to their phylogenetic conservation, so that conserved bases had less chance to be mutated. The resulting “positive” sequences were compared with a similar number of other TF-ChIP “negative” sequences not presenting either SRE or NFY in close proximity. The model performed well, since it was able to correctly classify the 96% of the training and validation set (S2 Table). After the training step, the model ability to predict of SRE-NFY sequences was tested in all the known gene promoters, by splitting the first five hundred bases of every promoter in a series of overlapping sequences of 100 bp with a shift of 20 bp that were fed into the model in prediction mode. The genes were then grouped by biological process according to the GO term classification, and odds ratios were calculated for GO clusters enriched in gene promoters containing the searched SRE-NFY pattern (Table 1). Interestingly, cholesterol synthesis was not the top enriched biological process, since the GO groups “cholesterol biosynthetic process” (GO:0006695) and “regulation of cholesterol biosynthetic process” (GO: 0045540) were at 3^rd^ and 6^th^ place respectively (Table 1). The most enriched GO term was “mismatch repair” (GO:0006298), containing fourteen genes (45% of the total genes) positive for a predicted SRE-NFY pattern. These data suggest that the mismatch repair biological process is somehow related to cholesterol signaling. Patients affect by Lynch syndrome are characterized by mutations in the genes of the mismatch repair GO group (*MLH1*, *MSH2*, *MSH6*, *PMS2*, *EPCAM*), determining increased risk of cancer, and this risk is higher in presence of elevated cholesterol levels [14]. Then, it could be speculated that the increased cancer risk of some patients with Lynch syndrome might be related to the further repression of the mismatch repair related genes by intracellular cholesterol via SRE modulation.

Also, the regulation of the RNA metabolic process GO term (GO:0016070) presented an excess of genes containing the SRE-NFY patterns according to the CNN model. Among the fifteen positive promoters (40% of the total genes in the group), six of them contained SRE binding sites and seven of them contained NFY binding sites according to ChIP experiments. The model predicted SRE-NFY patterns in five genes without any SRE or NFY binding site detected by ChIP experiments. Among the genes of the RNA metabolic process, nine of them encode for heterogeneous nuclear ribonucleoproteins, that have been linked to cholesterol metabolism in different reports. The *HNRNPA1* gene has been demonstrated to regulate the *HMGCR* gene expression, modulating cholesterol synthesis [15], *HNRNPA2B1* regulated the expressions of cyp7a1 and abca1 in Hepatocytes [16], and *HNRNPD* down-regulated the LDL-receptor [17]. *NCL* is another gene of this group, encoding for nucleolin, and its suppression in HeLa cells determined a decrease in intracellular cholesterol content [18]. These evidences show that the proposed CNN model is able to identify cholesterol responsive patterns in genes not directly related to cholesterol metabolism. In some cases, the CNN was activated by sequences that did not present SRE or NFY binding sites either according to ChIP experiments or to the JASPAR prediction tool. Sometimes the model was activated by putative SP-1 or SP-1-like binding sites (like KLF-5), which are known to flank the SRY-NFY couple in steroidogenic promoters [8, 11]. Other interesting cases are those in which a SRE binding site was identified by ChIP experiments, but without any putative SRE sequence detected by JASPAR. Though we analyzed only few promoters in detail, we found that, in absence of SRE or NFY sequences, the model seemed activated repeatedly by sequences identified as SP-1, RXR-A, ZNF263 or ZNF423 (S6–S8, S10 and S11 Figs). ZNF263 is reverse-complementary to SP-1, which is characterized by the CCCxCCC pattern, that also characterizes the KLF-like binding site (S12 Fig). RXR-A and ZNF423 present complex patterns that could not be associated with known SRE, NFY or SP1 -like sequences.

In conclusion, we generated and trained a CNN model able to predict the occurrence of cholesterol responsive SRE, NFY or SRE-NFY couples in gene promoters, and we evaluated a strategy that allowed to identified biological processes that were not apparently correlated with cholesterol metabolism but that resulted enriched in genes regulated by cholesterol. More, we partially decrypted the model output, confirming that known SRE, NFY and SP-1 sequences were able to activate the model, but we also found that other sequences might be relevant in promoter regions able to bind SREBPs, but without canonical SRE sequences found.

The main limit of the study was not designed to confirm “in vitro” the binding SREBPs to their putative targets. The study was able to confirm the presence of SREBPs binding sites in genes in which the binding site were already identified, but further molecular studies are required to prove that the novel putative target sequences propose in this study are effectively relevant to the binding of SREBPs to gene promoters. Further studies might expand this issue and help to identify novel atypical TF consensus sequences.

## Supporting information

S1 Fig Distribution of SRE <> NFY peaks distances (ENCODE TF-ChIP) in peaks containing a putative SRE-NFY site. (TIF)

S2 Fig CNN 1D model. (TIF)

S3 Fig Evaluation of the *SQLE* promoter by the CNN 1-D model. (ZIP)

S4 Fig Evaluation of the *CCNT1* promoter by the CNN 1-D model. (ZIP)

S5 Fig Evaluation of the *STARD4* promoter by the CNN 1-D model. (ZIP)

S6 Fig Evaluation of the *IDI1* promoter by the CNN 1-D model. (ZIP)

S7 Fig Evaluation of the *POLD2* promoter by the CNN 1-D model. (ZIP)

S8 Fig Evaluation of the *NCL* promoter by the CNN 1-D model. (ZIP)

S9 Fig Evaluation of the *HNRNPA2B1* promoter by the CNN 1-D model. (ZIP)

S10 Fig Evaluation of the *PCPB2* promoter by the CNN 1-D model. (ZIP)

S11 Fig Evaluation of the *RPA2* promoter by the CNN 1-D model. (ZIP)

S12 Fig Transcriptions Factors binding sites JASPAR representation. (TIF)

S1 Table List of ENCODE transcription factors binding sites from ENCODE ChIP experiments. (PDF)

S2 Table CNN-1D model performance. (PDF)

S1 File (PDF)
