# Supplementary figures and images for: DeepSRE: Identification of sterol responsive elements and nuclear transcription factors Y proximity in human DNA by Convolutional Neural Network analysis
Source: PLoS One. 2021 Mar 4;16(3):e0247402. doi: 10.1371/journal.pone.0247402 (PMC7932541; doi:10.1371/journal.pone.0247402)

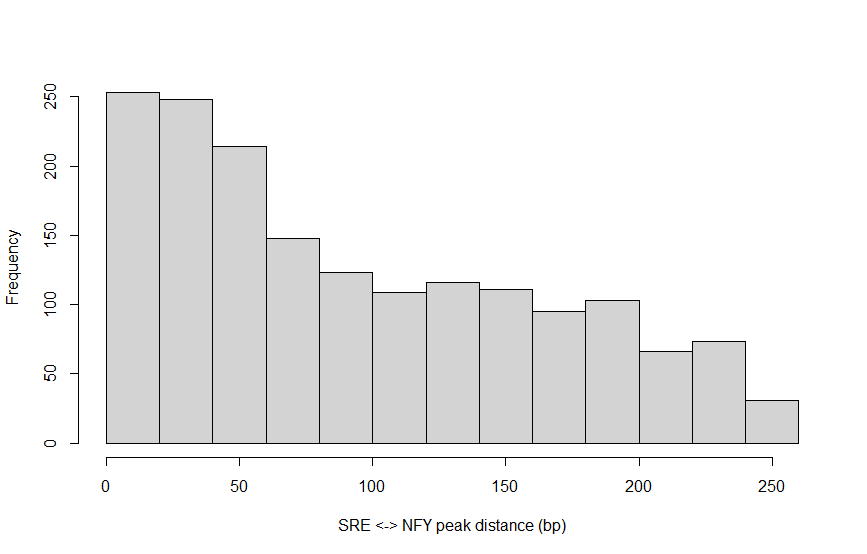

Supplement: S1 Fig — (TIF) [file pone.0247402.s001.tif]

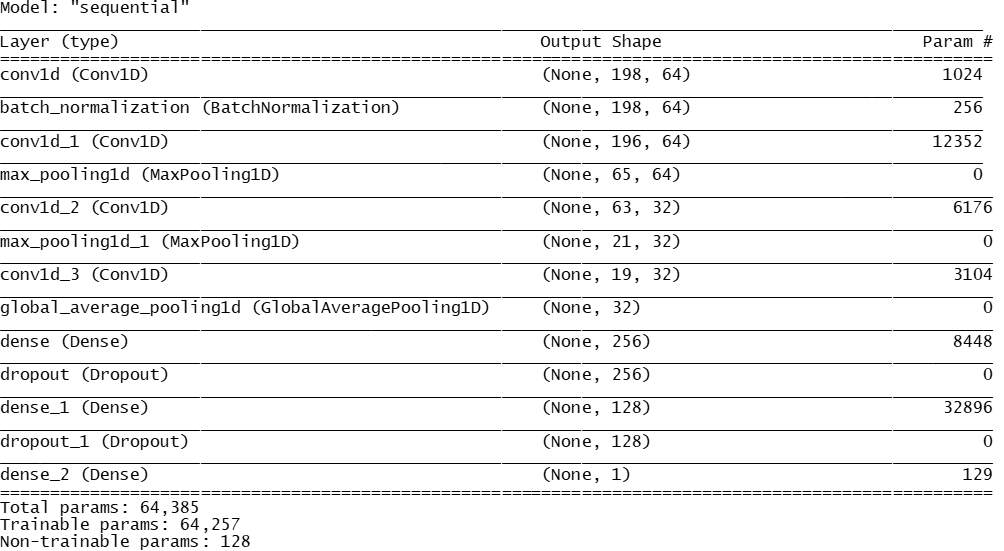

Supplement: S2 Fig — (TIF) [file pone.0247402.s002.tif]

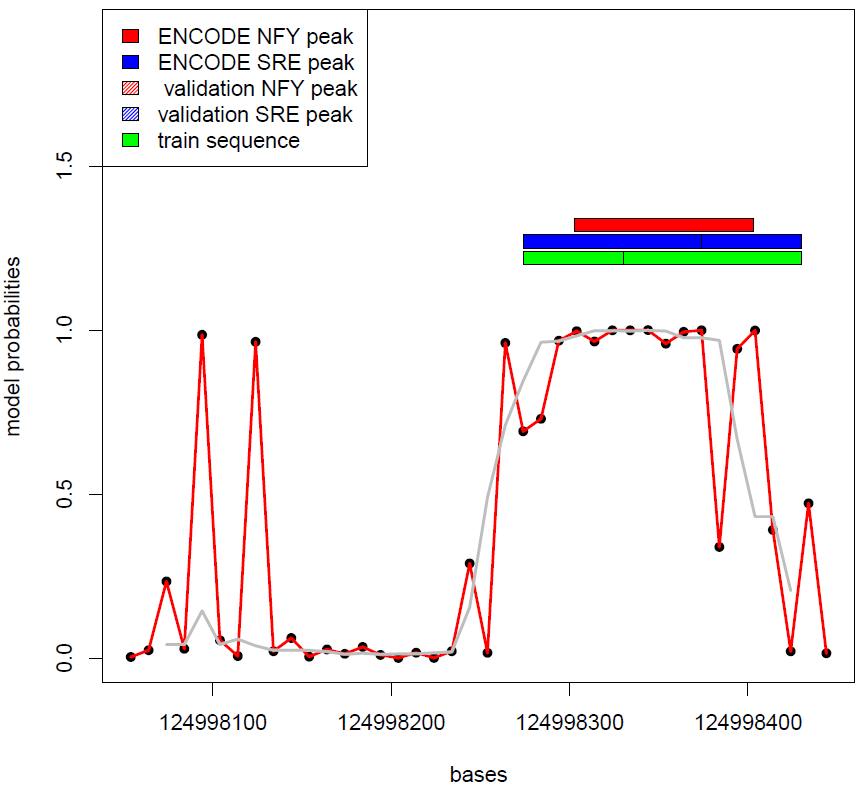

Supplement: S3 Fig — (ZIP) [file pone.0247402.s003.zip › S3A_Fig.tif]

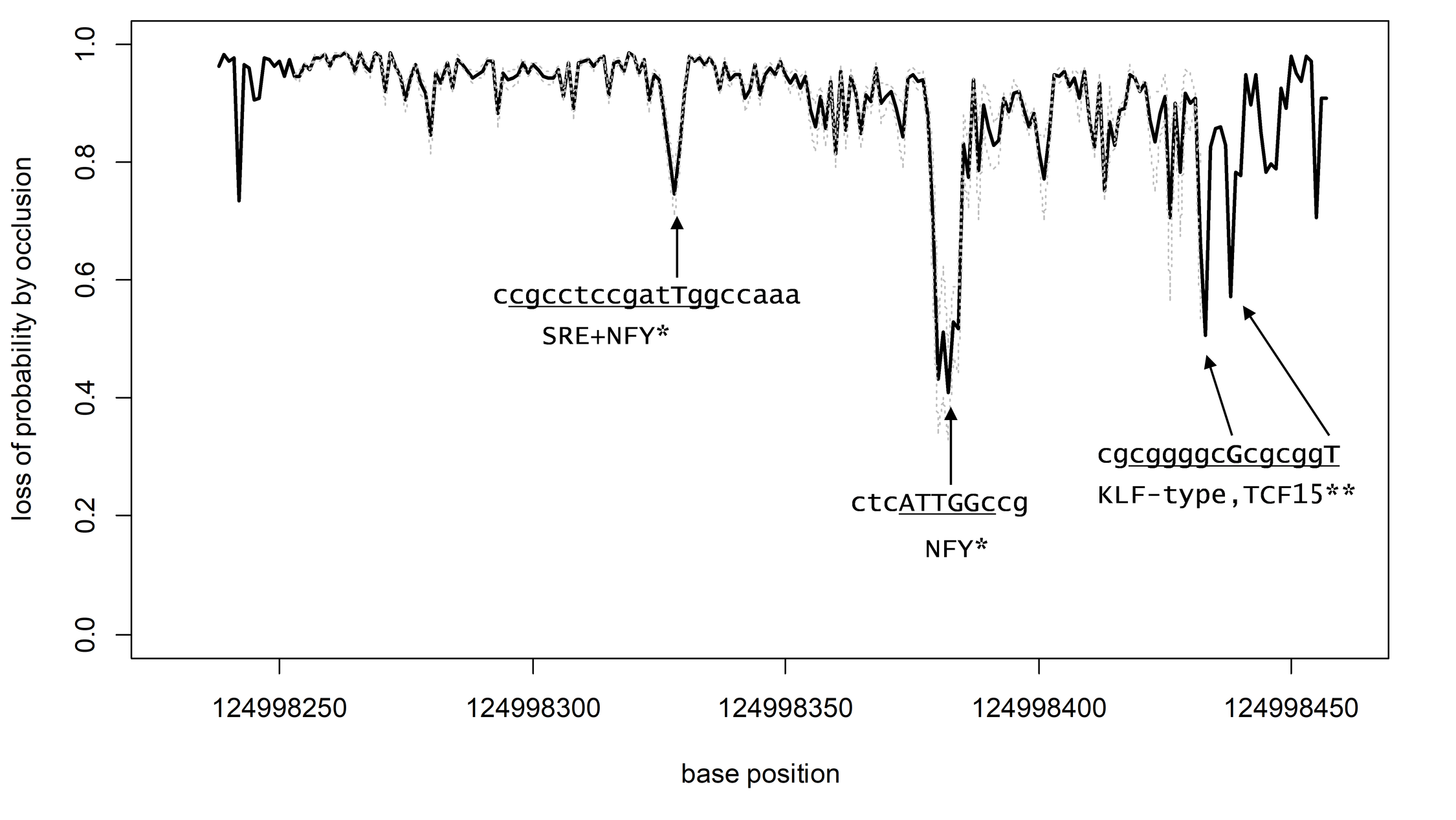

Supplement: S3 Fig — (ZIP) [file pone.0247402.s003.zip › S3B_Fig.tif]

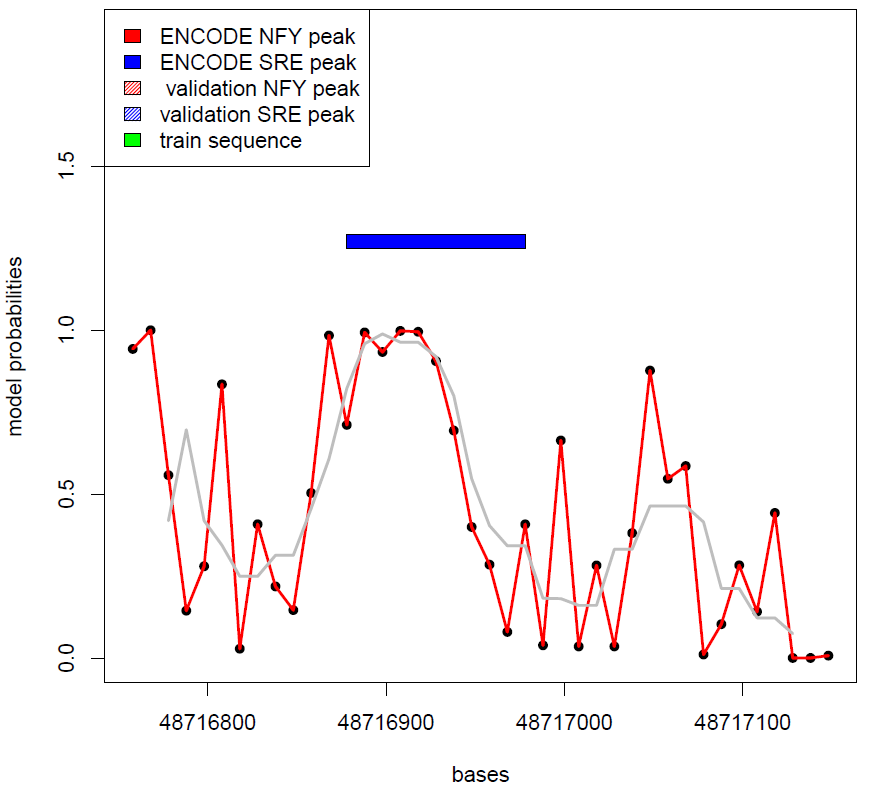

Supplement: S4 Fig — (ZIP) [file pone.0247402.s004.zip › S4A_Fig.tif]

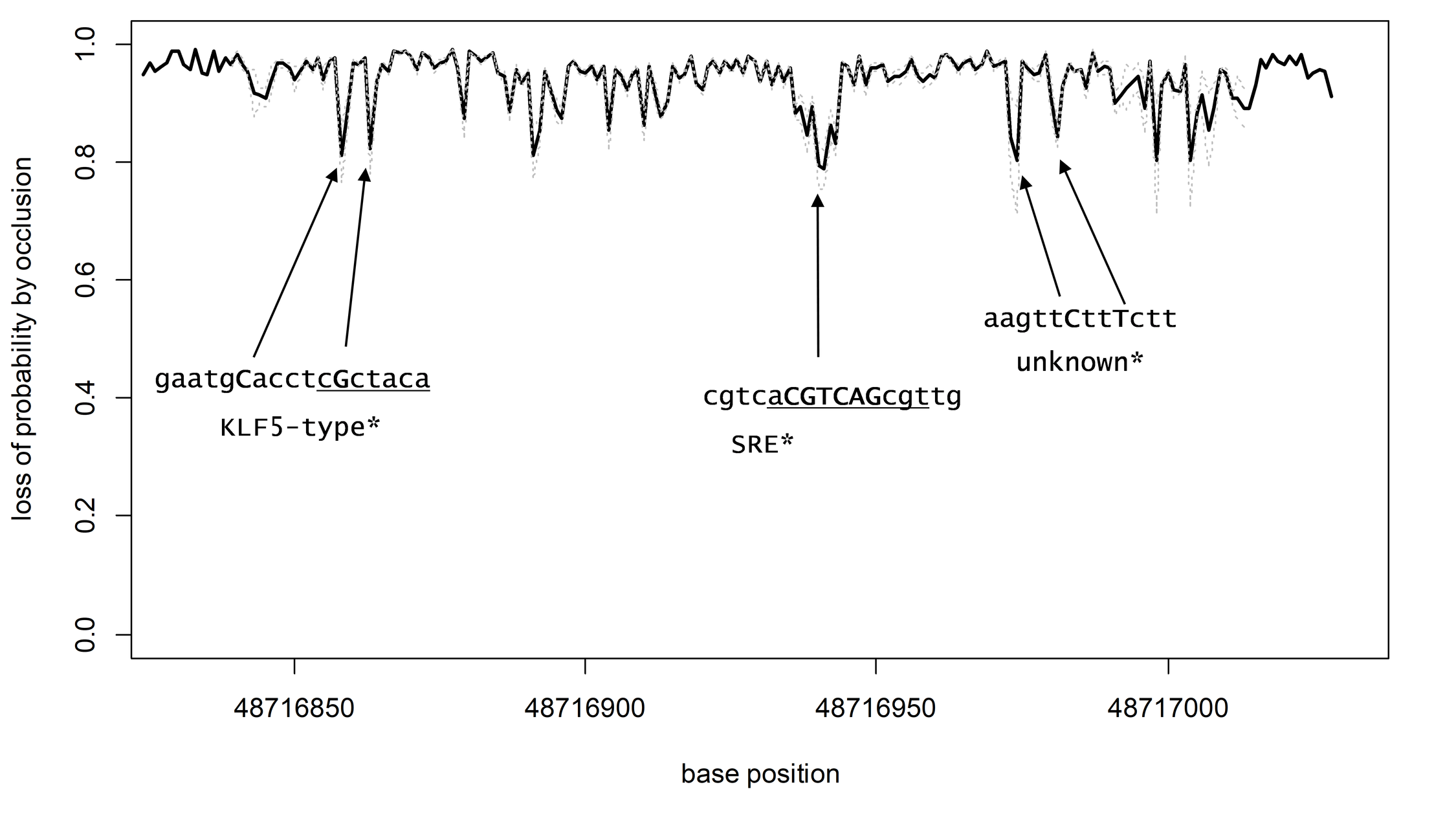

Supplement: S4 Fig — (ZIP) [file pone.0247402.s004.zip › S4B_Fig.tif]

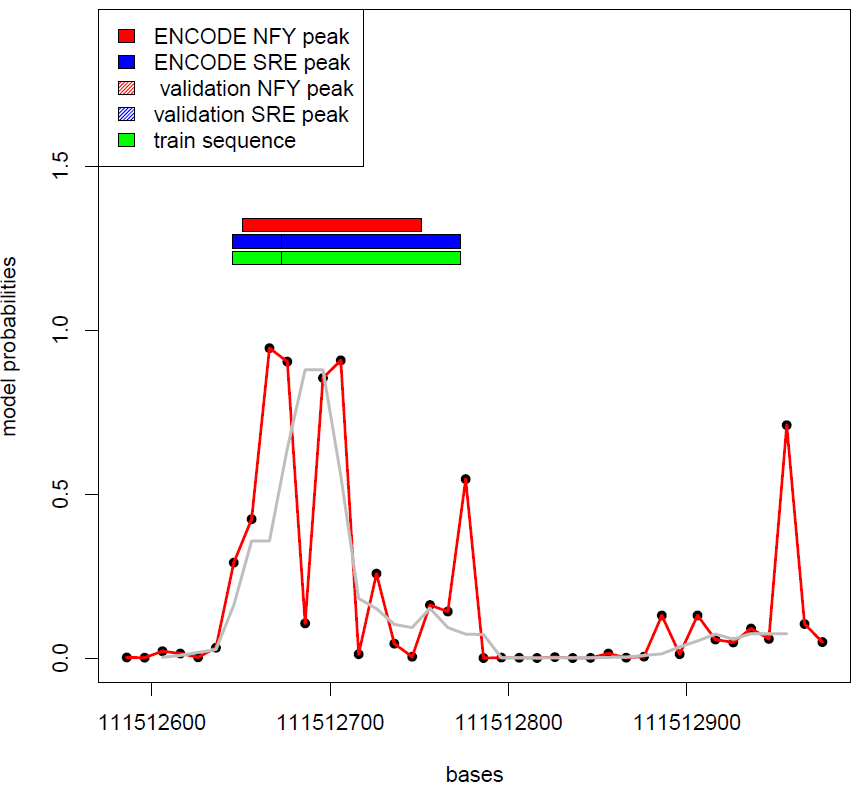

Supplement: S5 Fig — (ZIP) [file pone.0247402.s005.zip › S5A_Fig.tif]

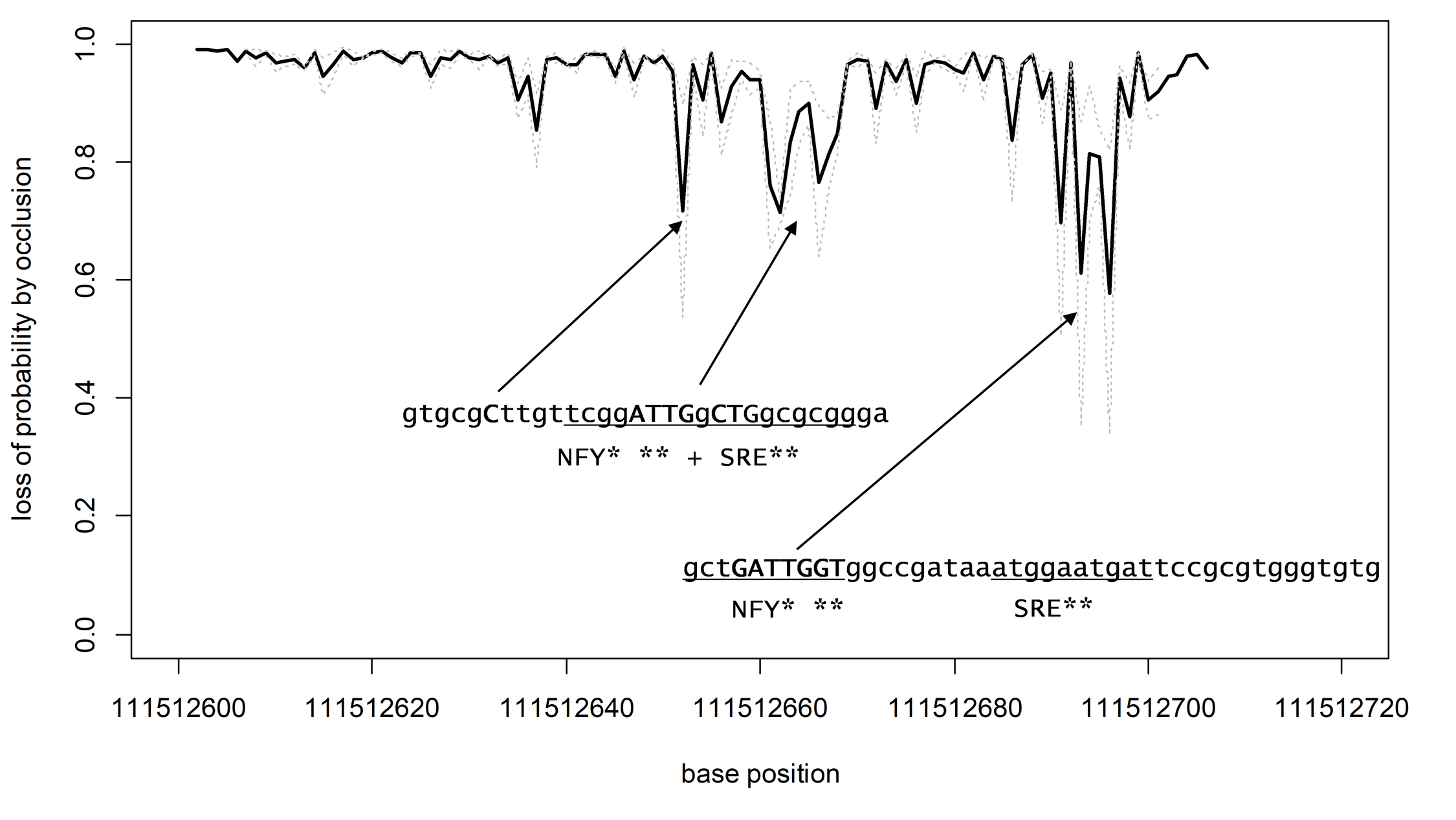

Supplement: S5 Fig — (ZIP) [file pone.0247402.s005.zip › S5B_Fig.tif]

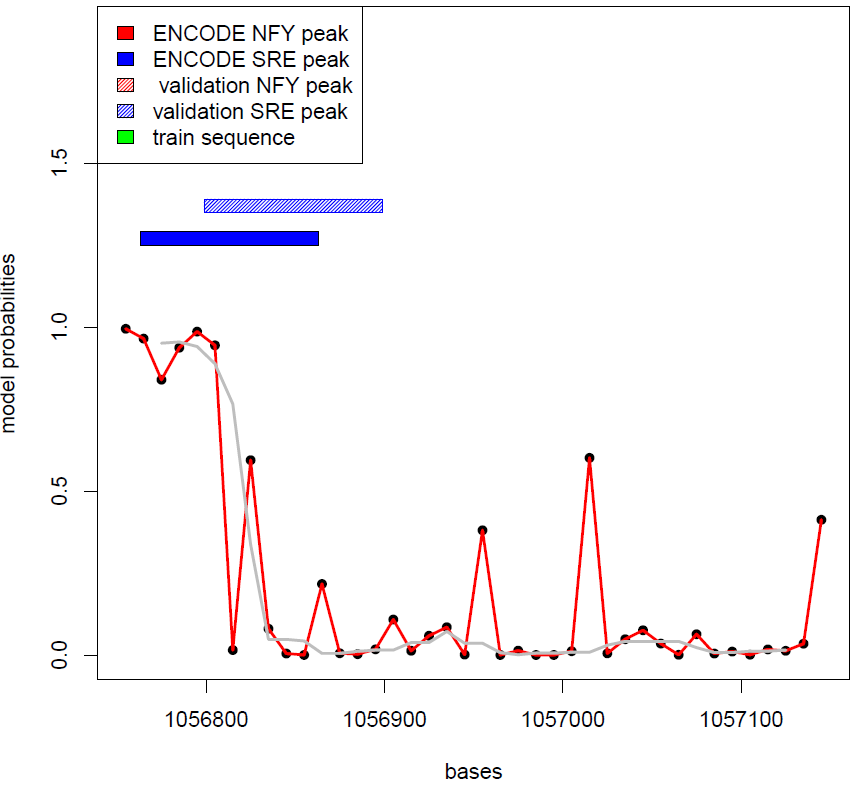

Supplement: S6 Fig — (ZIP) [file pone.0247402.s006.zip › S6A_Fig.tif]

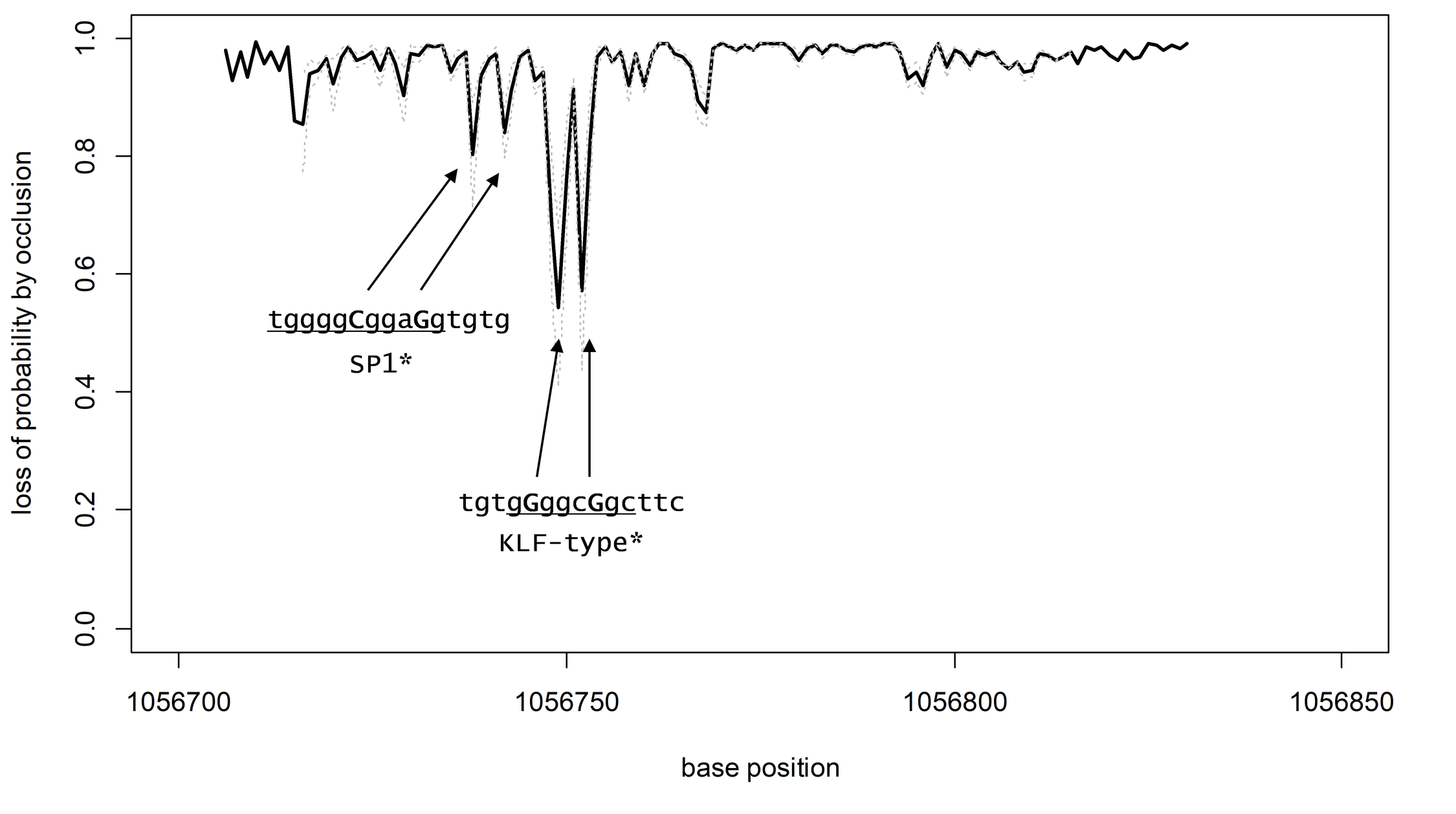

Supplement: S6 Fig — (ZIP) [file pone.0247402.s006.zip › S6B_Fig.tif]

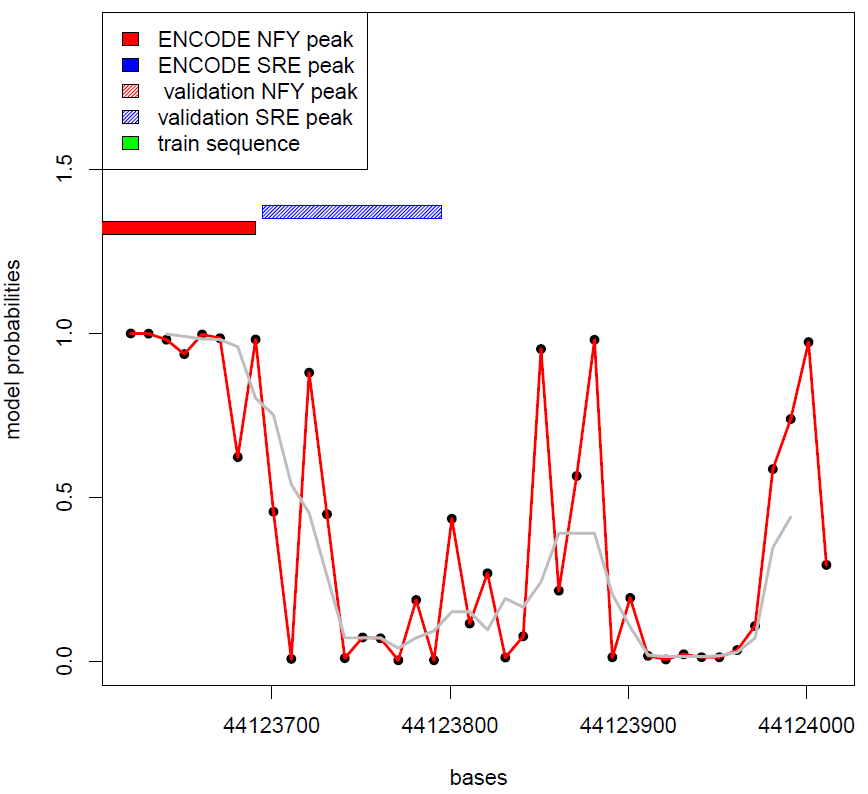

Supplement: S7 Fig — (ZIP) [file pone.0247402.s007.zip › S7A_Fig.tif]

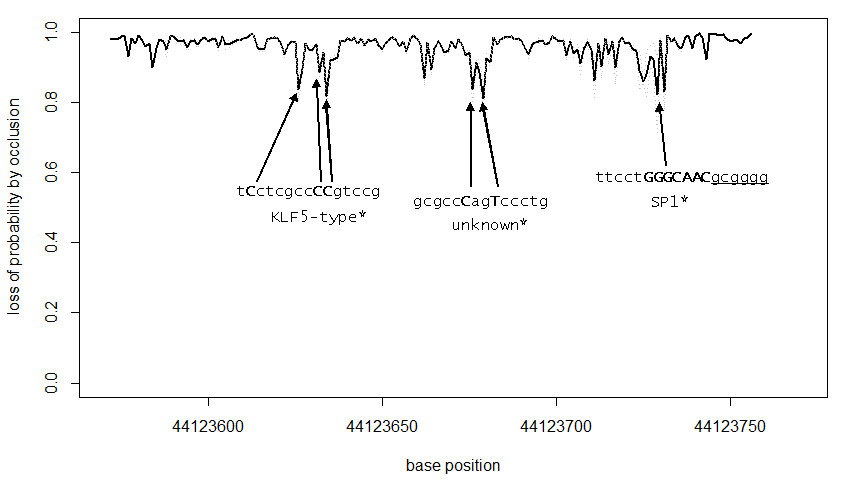

Supplement: S7 Fig — (ZIP) [file pone.0247402.s007.zip › S7B_Fig.tif]

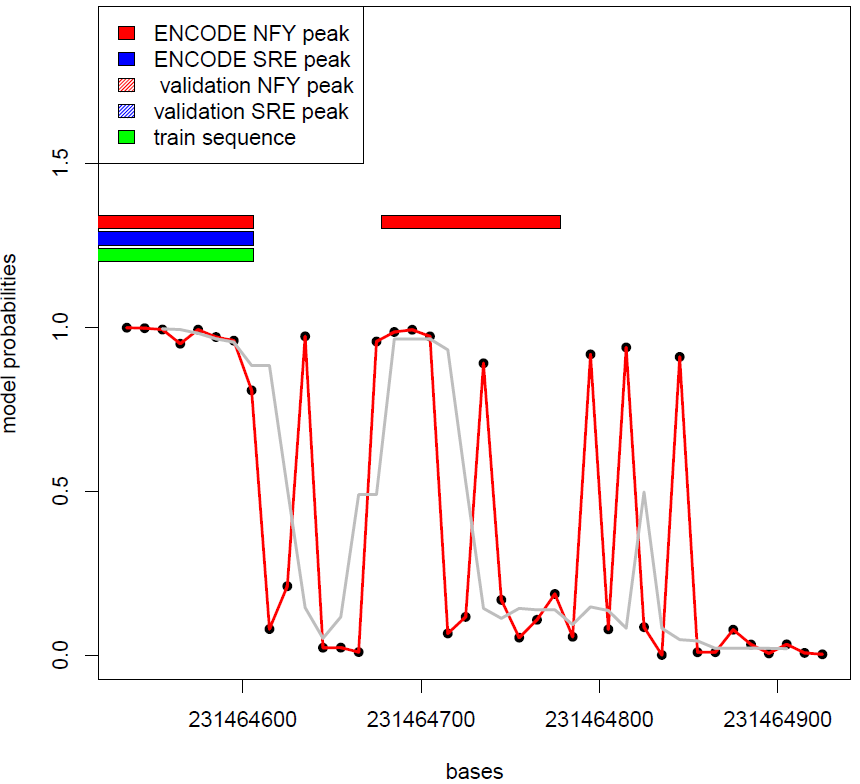

Supplement: S8 Fig — (ZIP) [file pone.0247402.s008.zip › S8A_Fig.tif]

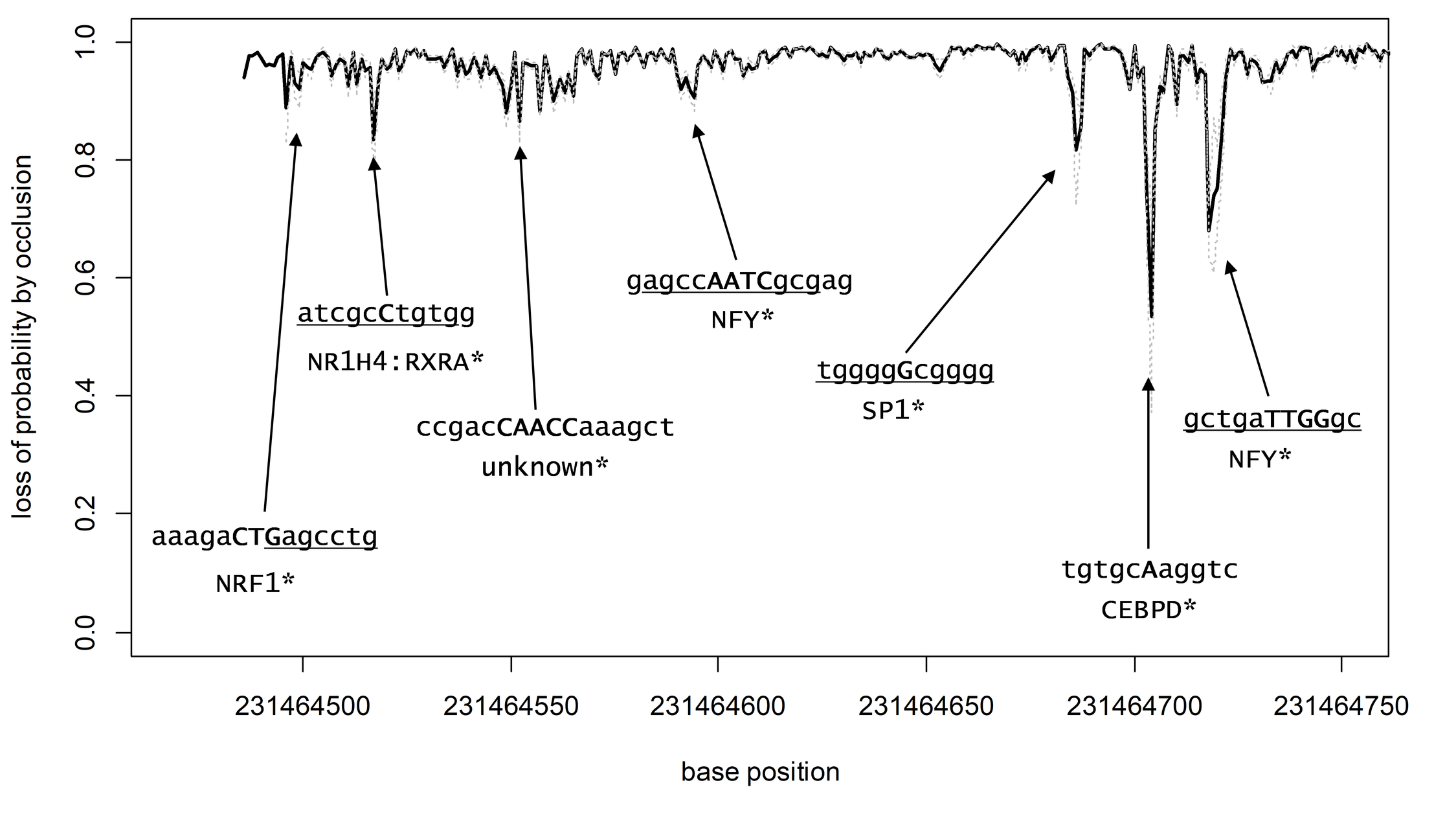

Supplement: S8 Fig — (ZIP) [file pone.0247402.s008.zip › S8B_Fig.tif]

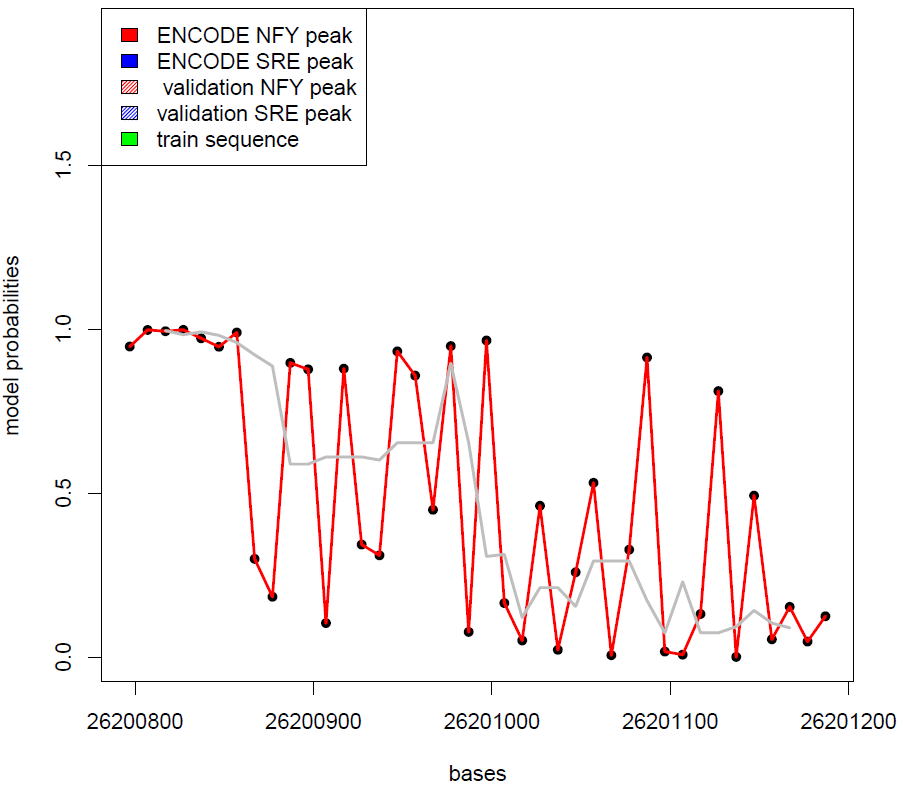

Supplement: S9 Fig — (ZIP) [file pone.0247402.s009.zip › S9A_Fig.tif]

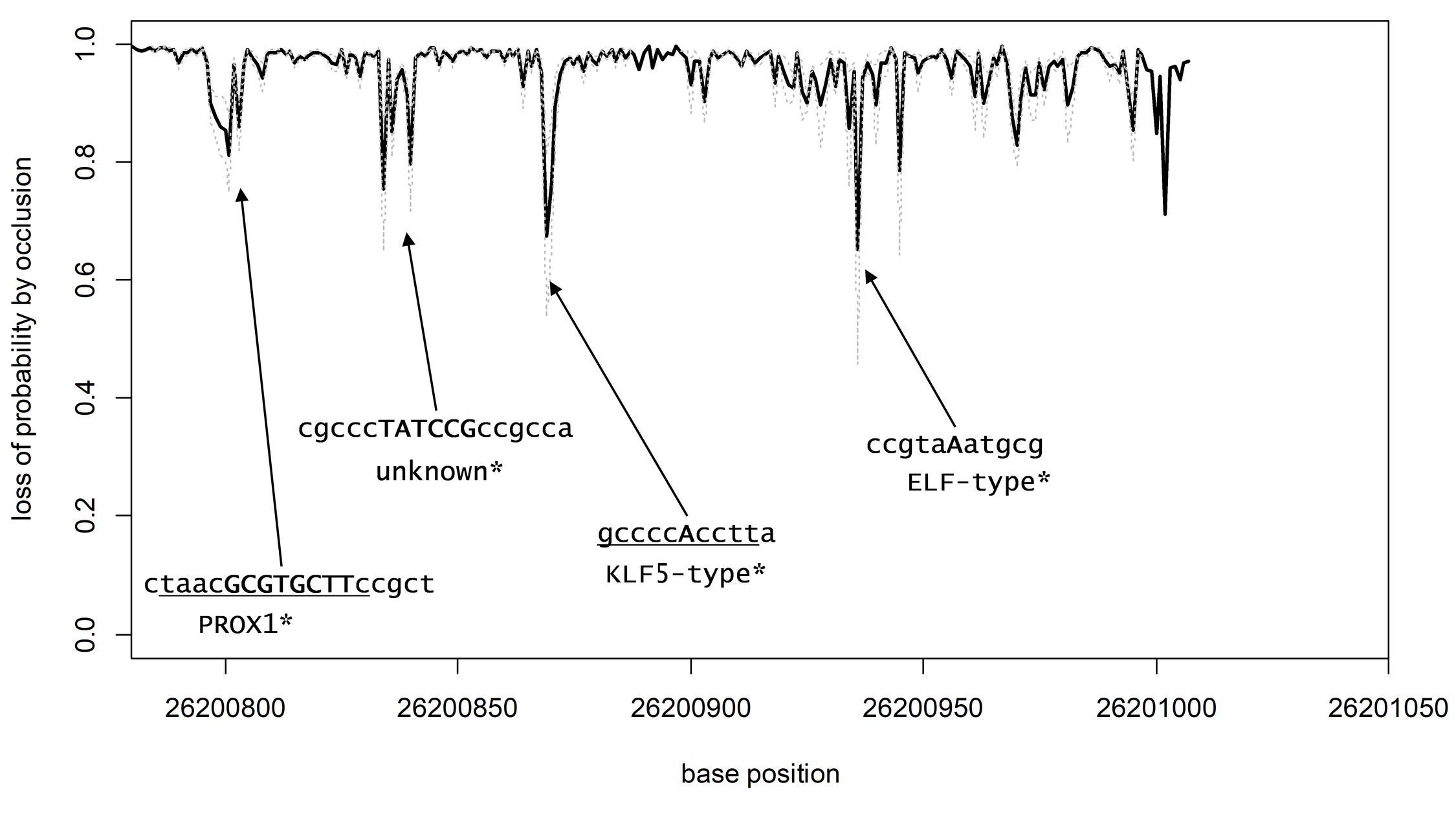

Supplement: S9 Fig — (ZIP) [file pone.0247402.s009.zip › S9B_Fig.tif]

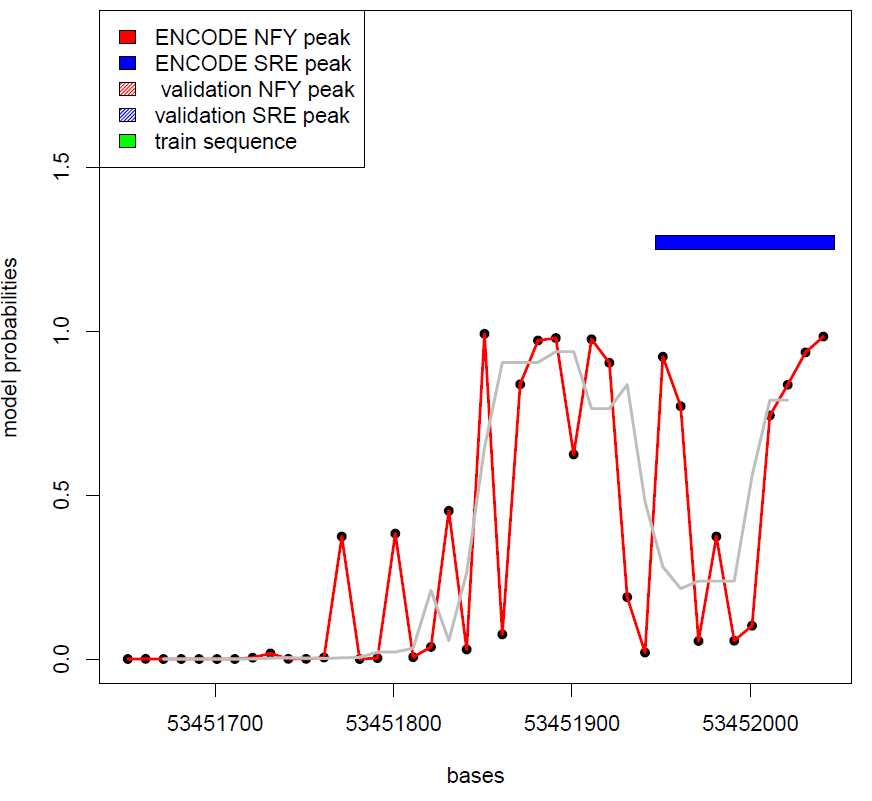

Supplement: S10 Fig — (ZIP) [file pone.0247402.s010.zip › S10A_Fig.tif]

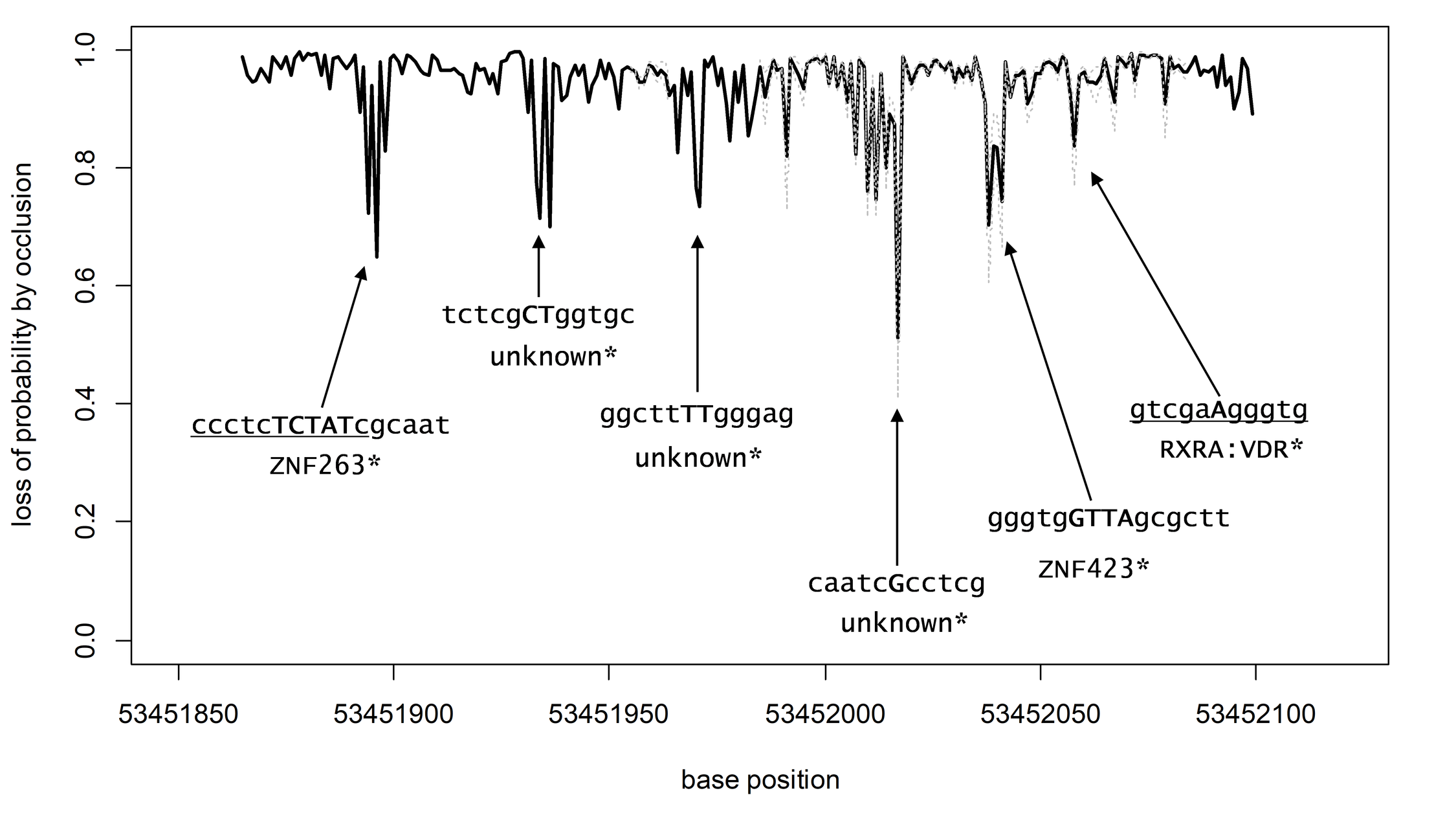

Supplement: S10 Fig — (ZIP) [file pone.0247402.s010.zip › S10B_Fig.tif]

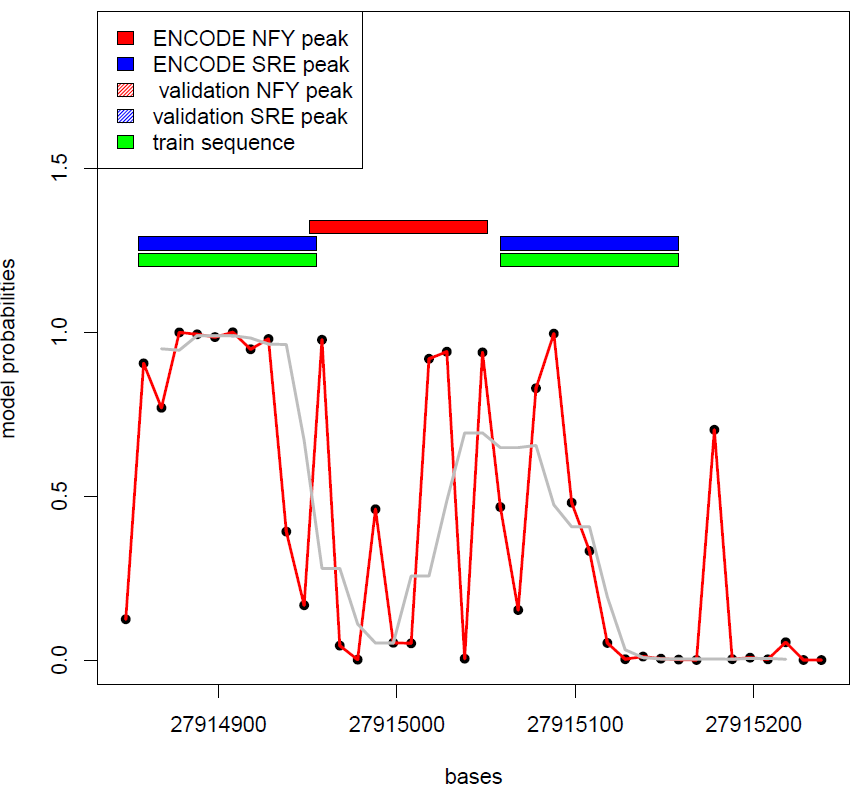

Supplement: S11 Fig — (ZIP) [file pone.0247402.s011.zip › S11A_Fig.tif]

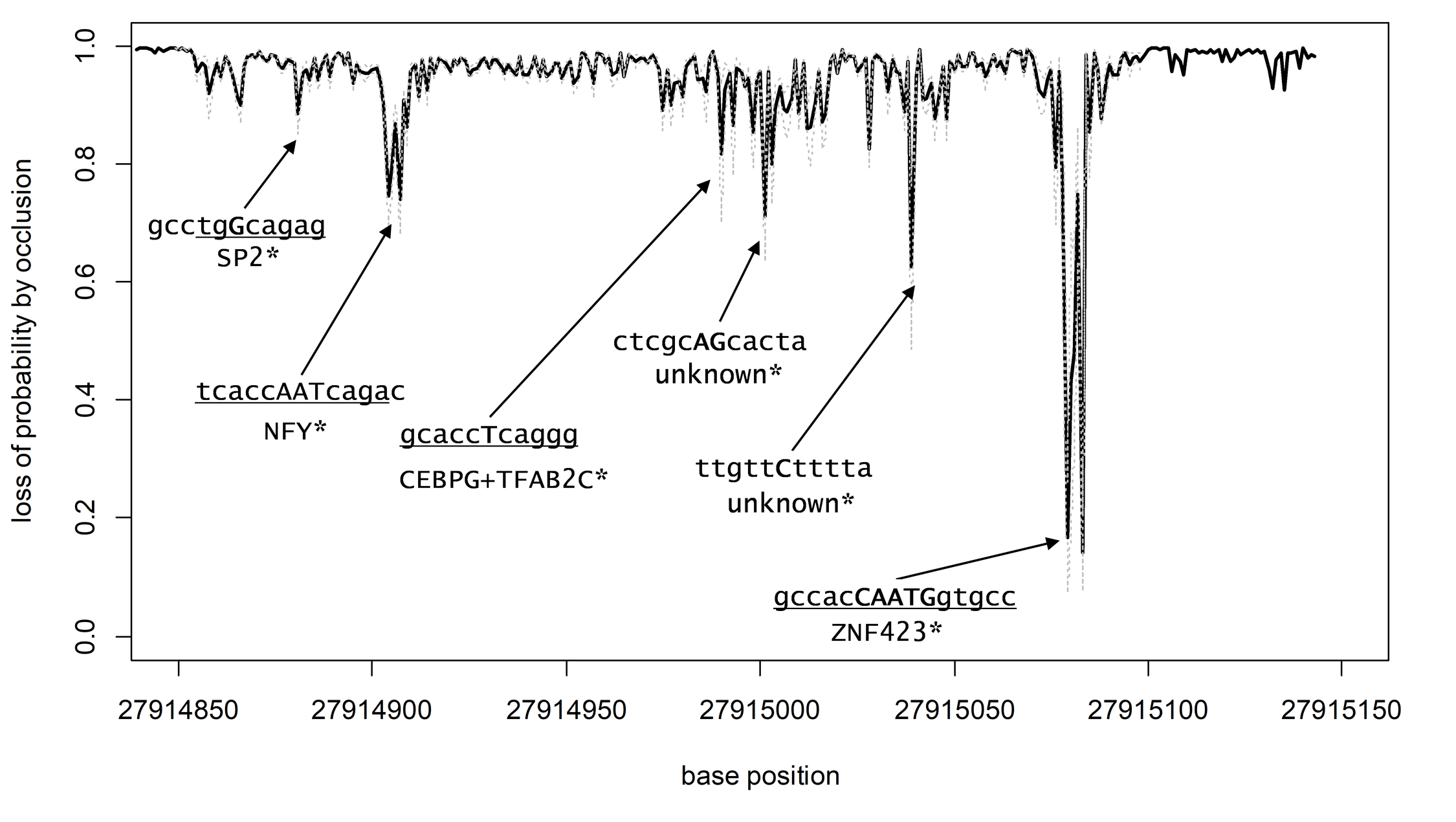

Supplement: S11 Fig — (ZIP) [file pone.0247402.s011.zip › S11B_Fig.tif]

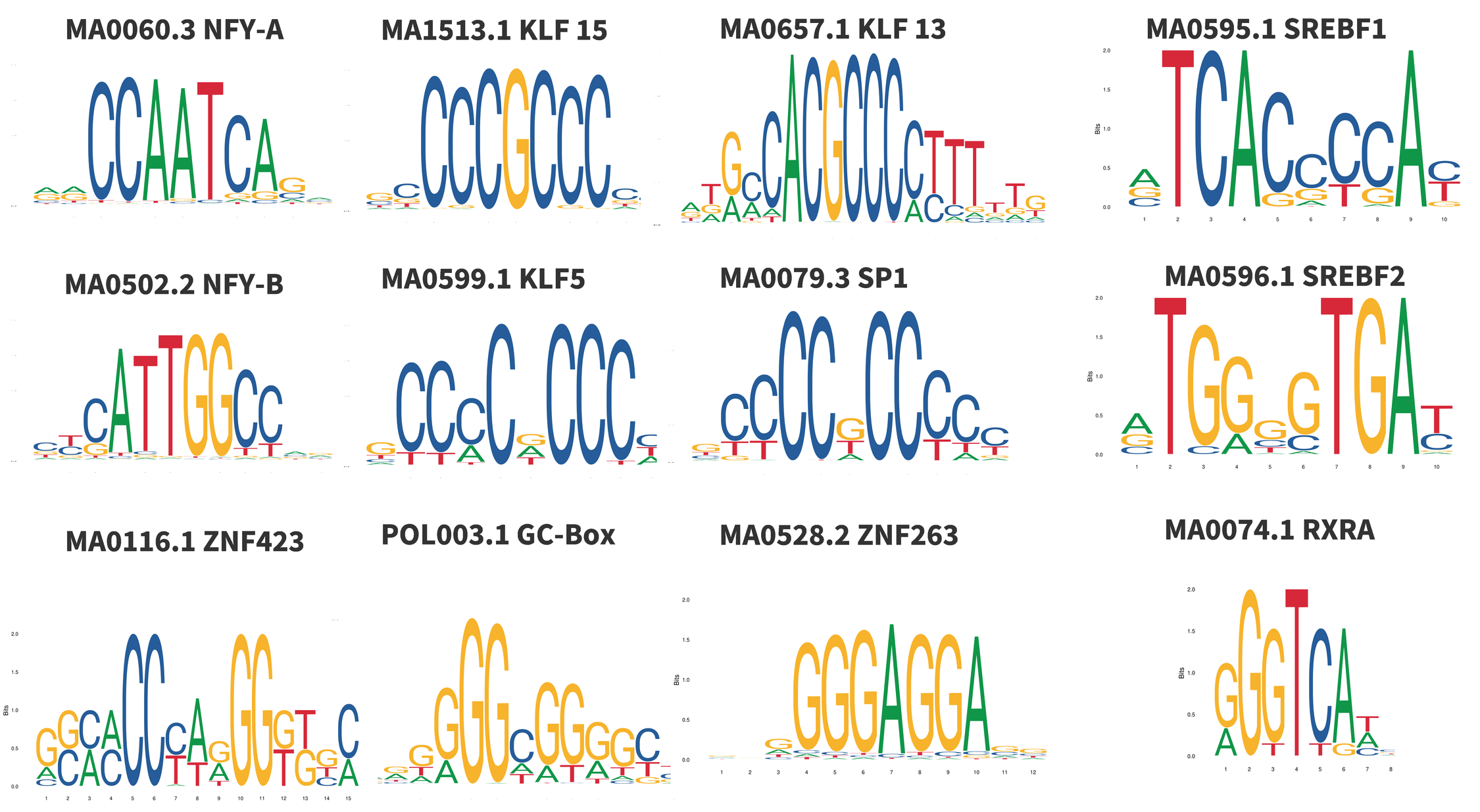

Supplement: S12 Fig — (TIF) [file pone.0247402.s012.tif]
