# Supplementary material for: DeepSRE: Identification of sterol responsive elements and nuclear transcription factors Y proximity in human DNA by Convolutional Neural Network analysis
Source: PLoS One. 2021 Mar 4;16(3):e0247402. doi: 10.1371/journal.pone.0247402 (PMC7932541; doi:10.1371/journal.pone.0247402)
Supplement: S1 Table — (PDF) [file pone.0247402.s013.pdf]

Supplemental Table 1: List of ENCODE trascription factors binding sites from ENCODE ChIP experiments.

Primary Table	Cell type	Transcript.Factor	Row Count
**Positive peaks (SRE or NFY)**			
encTfChipPkENCFF624DDK	A549	SREBF1	3429
encTfChipPkENCFF483YCC	A549	SREBF2	838
encTfChipPkENCFF278GJK	GM12878	NFYA	1143
encTfChipPkENCFF510NDO	GM12878	NFYB	12736
encTfChipPkENCFF777MYW	K562	SREBF1	2950
**Control peaks**			
encTfChipPkENCFF297HAX	A549	FOXA1	33874
encTfChipPkENCFF907WHF	A549	PHF8	17048
encTfChipPkENCFF807XMX	A673	EZH2	7986
encTfChipPkENCFF096XRG	GM12878	ASH2L	7059
encTfChipPkENCFF035GFS	GM12878	E4F1	6443
encTfChipPkENCFF978BBL	K562	IRF1	12351
encTfChipPkENCFF262TMM	K562	MITF1	34723
encTfChipPkENCFF168JLI	liver	FOXA2	25164
