# Supplementary material for: DeepSRE: Identification of sterol responsive elements and nuclear transcription factors Y proximity in human DNA by Convolutional Neural Network analysis
Source: PLoS One. 2021 Mar 4;16(3):e0247402. doi: 10.1371/journal.pone.0247402 (PMC7932541; doi:10.1371/journal.pone.0247402)
Supplement: S2 Table — (PDF) [file pone.0247402.s014.pdf]

Supplemental Table2: CNN-1D Model Performance

Model evaluate: model accuracy = 0.963, model loss = 0.00992

A.U.C(95% confidence intervals) =0.981 (0.980-0.981)

Best A.U.C. threshold = 0.760

Model prediction:

	**Predicted negatives**	**Predicted positives**	**Totals**
**True negative**	130217	6085	136302
**True positive**	5642	125759	131401
**Total**	135859	131844	267703

Model performance (Yardstick package*)

Accuracy = 0.956, Precision = 0.954, Recall = 0.957, F1 score = 0.955
