# Supplementary material for: DeepSRE: Identification of sterol responsive elements and nuclear transcription factors Y proximity in human DNA by Convolutional Neural Network analysis
Source: PLoS One. 2021 Mar 4;16(3):e0247402. doi: 10.1371/journal.pone.0247402 (PMC7932541; doi:10.1371/journal.pone.0247402)
Supplement: S1 File — (PDF) [file pone.0247402.s015.pdf]

DeepSRE

Introduction

DeepSRE is a procedure that uses Deep Learning to identify possible SRE-NFY co-occurrence in gene promoters. The Deep Learning model consist of a Convolutional Neural Network 1-D model that is trained on SRE and NFY putative sequences contained in peaks identified by Transcription Factor Chromatin Immuno Precipitation (TF-ChIP) experiments. The peaks are identified by chromosomal coordinates listed in the ENCODE network database interrogated by the UCSC genome browser Table tool.

The procedure do not represent a fully operational software, but it is a simple list of R language routines that must be executed sequentially within the Rstudio environment to produce the desired output. Working directories, working files requires by the procedures, are to be set manually, so an "a-priori" knowledge of R language and RStudio functionalities are also required.

DeepSRE requires Reticulate, Keras and Tensorflow packages to work. Anaconda 3 was used to create a R python environment named "r-tensorflow", that contains Tensorflow 1.14. The program has not been tested in Tensorflow 2.0. The complete procedure to install Keras and Tensorflow in RStudio is explained in the dedicated "R interface to Keras" site at https://keras.rstudio.com/. An intermediate knowledge of Keras is also suggested.

Directory structure

The procedure requires a series of files located in appropriate directories to work. The scheme of the directories is presented in Figure 1.

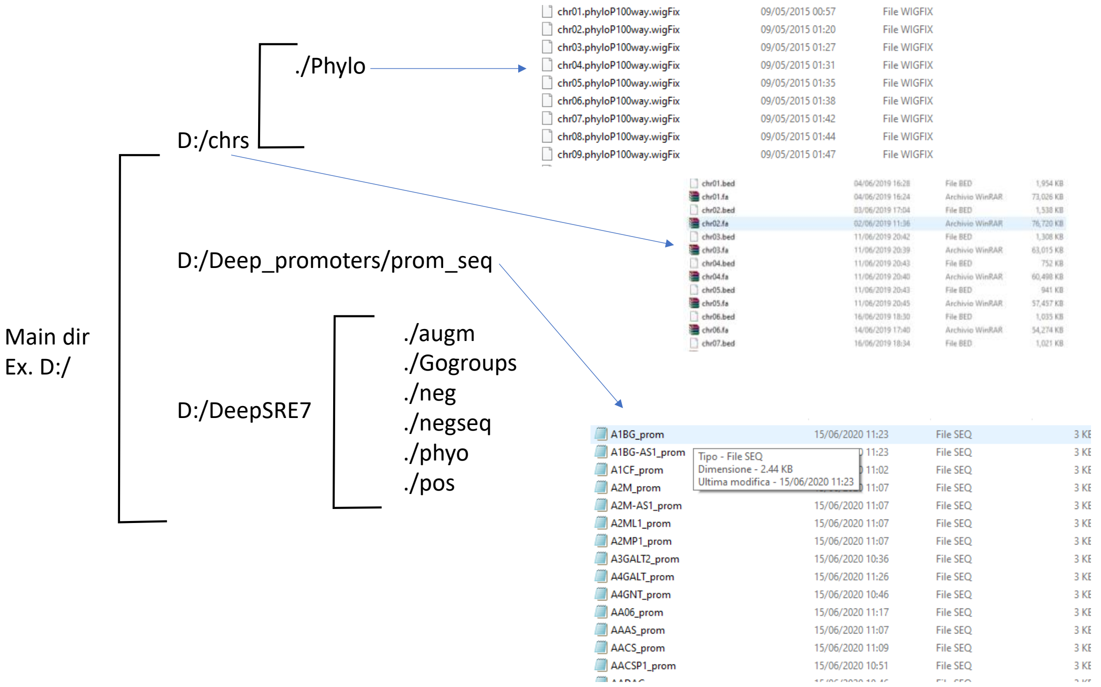

The "chrs" dir list all the chromosomal sequences in FASTA and all transcripts start-stop in BED format, all obtained from https://hgdownload.soe.ucsc.edu/goldenPath/hg38/chromosomes/, UCSC genome resources. The "Phylo" subdirectory contains the list of phylogenetic conservation scores in wigFIX format, from http://hgdownload.cse.ucsc.edu/goldenpath/hg38/phyloP100way/. The first 500 bases of each promoter are extracted and stored in the "Deep_promoters/prom_seq" dir.

The "DeepSRE7" is the main dir. The content of subdirs is shown in Figure 2

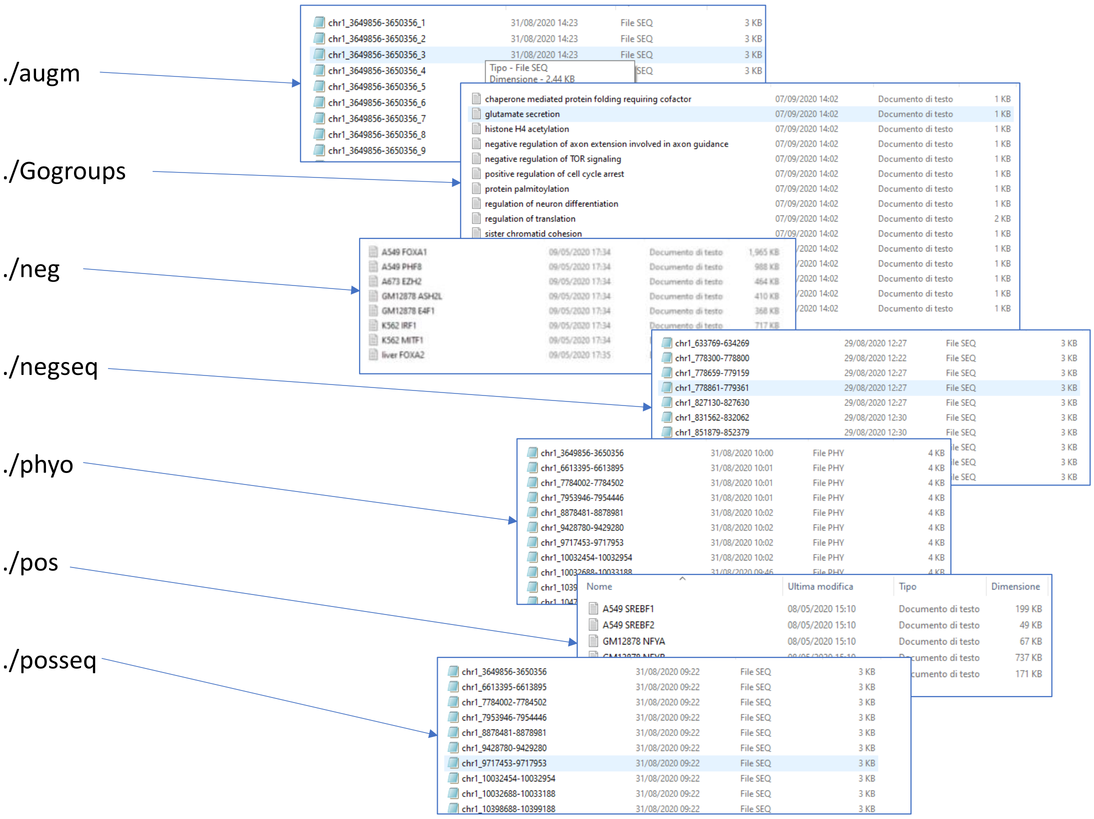

All of the subdirs are populated during the execution of the program with the exception of the ./neg and ./pos dirs., that present the TFChIP peaks data obtained from http://genome.ucsc.edu/ and converted in text format. ./neg are control TFChIP peaks, ./pos are SRE and NFY peaks.

Workflow.

The procedure is composed of a series of steps included in consecutive R files. The R files must be executed in order, from 1 to 9 as shown in Figure 3. Some files contain R functions to support the main files.

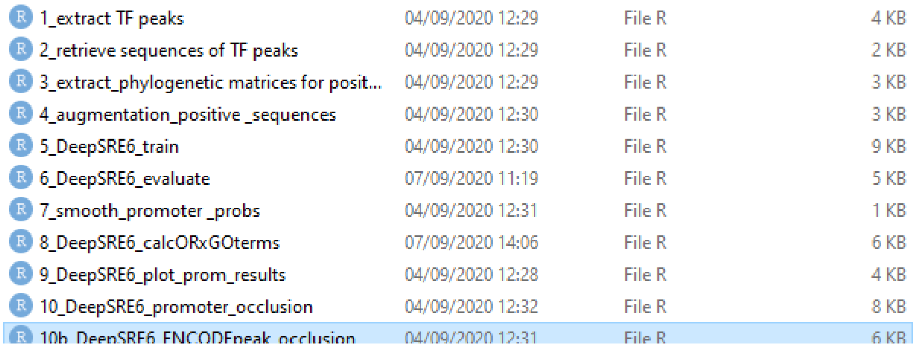

Steps 1 to 5

The first steps of the procedure are depicted in Figure 4. First, positive and negative TFChIP peaks are selected and stored in the ./neg and ./pos subdirs. Then peaks of SRE and NFY are extracted and compared to each other to check for a "SRE – 250 bp – NFY" couple where peaks are located within 250 bp of distance. Random peaks for other TF represent the negative controls, but the control peaks are checked for not containing any SRE peak within 1000 bp of distance. In that case the peak is discarded. Positive peaks are augmented by base mutations according to phylogenetic base conservation, meaning that most conserved bases are less prone to be mutated. Peaks are then converted to numerical matrix, reversed complemented and subjected to CNN1D training. These steps are performed by the 1 to 5 R files.

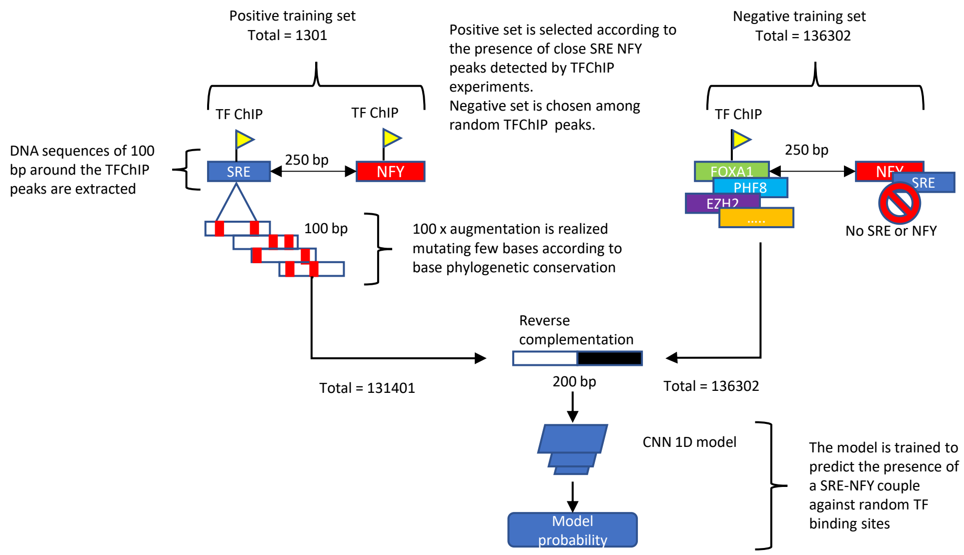

1_extract TF peaks

This procedure extract both negative and positive TF peaks locations, check for SRE-NFY couples and clean the negative TF peaks. Positive SRE-NFY couples are stored in the "ENCODE_SREBF&NFY_peak.txt" file, while negative peaks in the "ENCODE_negative_peak.txt" file.

2_retrieve sequences of TF peaks

This file recover the peak info from the above files and retrieve fragments of DNA sequence of 500 bp around the peaks and store them in the ./negseq and ./posseq dirs.

3_extract_phylogenetic matrices for positive sequences

This routine load the positive peaks DNA coordinates and retrieves the corresponding phylogenic scores from the D:/chrs/Phylo files in wigFix format and, for each .seq file, saves the corresponding .phy file in the ./phylo subdir for augmentation procedure.

4_augmentation_positive _sequences

This is the augmentation procedure. It creates multiple copies of ./posseq sequences mutating the bases according to the inverse of the phylogenetic score, so that important conserved bases are not easily mutated. The augmented sequences are stored in the ./augm dir with a _[1..100].seq suffix.

5_DeepSRE6_train

This is the core of the procedure. A CNN1D model contained in the "SRE_models" file is loaded by keras. The sequence files are handled by a custom generator contained in the "DeepSRE6_generator" file. The generator loads the sequence, complements it and appends the reverse sequence to the forward sequence. Then, the generator converts the sequences to numerical matrices, hot encodes them and fed them to the model in batches of 512 samples. The model fitting is divided in three steps of decreasing epochs with and early callback procedure that stops the current step if no improvements are recorded after a predetermined number of epochs. The best fitting result is saved in .h5 format.

Steps 6 to 8.

These steps apply the trained model to the gene promoters in search of SRY-NFY couples. The idea shown in Figure 5 is to let the model predict what it considers a SRE-NFY iterating over 100-bp overlapping fragments obtaining by fractionating the first 500 bp of all the promoters contained in the "Deep_promoters" dir. Then all the positive promoters are defined as those with at leat 3 consecutive probabilities > 0.9. In the next steps the genes are grouped according to Gene Ontology terms expressing

the biological process (i.e. cholesterol synthesis, or DNA repair etc.) A procedure calculates if a GO group contains an excess of genes with a SRE-NFY patter detected by the model and it produces a Odds ratio with confidence intervals.

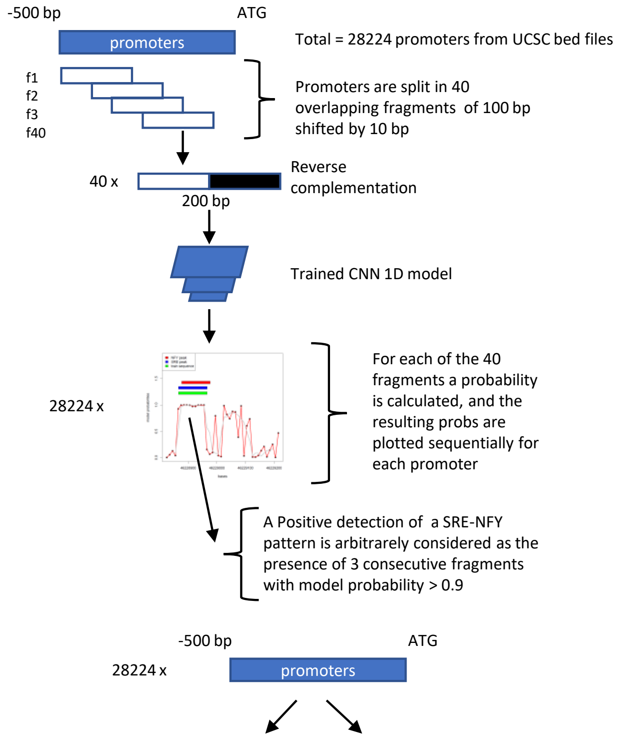

GO terms	SRE-NFY positives	SRE-NFY negatives	OR
total	3022	25202	-
mismatch repair	14	31	6.89
regulation of RNA metabolic	15	36	5.97
cholesterol biosynthetic process	16	40	5.58
positive regulation of cyclin-dependent protein serine/threonine kinase activity	12	31	5.28

Enrichemnt of Biological processes in promoters with detected SRE-NFY pattern

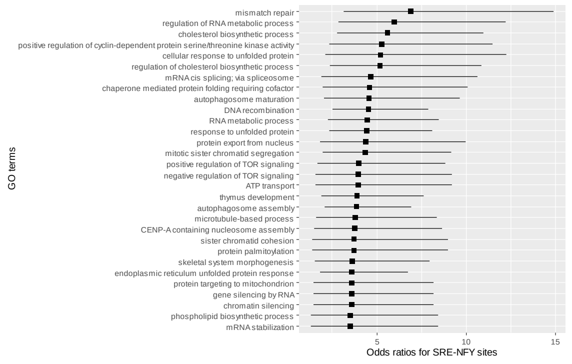

At the end of the process, 3022 promoters presented a positive detection of the SRE-NFY pattern. For each metabolic process, the positive/negative ratio is calculated and the odds ratios against all the other genes is calculated

6_DeepSRE6_evaluate

Every fragment is handled by the custom generator, as for the training sequences, and a model probability is produced. A matrix collects all the probabities of all the fragments of every promoter and stores it in the

“evalSRE7.csv” file. Since positive promoters used to train the model show at least three or more fragments with model probability > 0.9, the routine screens all the promoters for such pattern and stores it in the same “evalSRE7.csv” file.

7_smooth_promoter _probs

To avoid probability oscillations in consecutive fragments, the consecutive probabilities are smoothed following a custom rule. The procedure takes 5 consecutive probabilities, cut the 2 extremes and averages the remaining three, then the procedure steps forward by one probability and repeats the smoothing until the probability list is completed. The resulting smoothed probabilities are stored in the “averaged_mean_5_probs_SRE7.csv” file.

8_DeepSRE6_calcORxGOterms

This procedures retrieves the list of promoter probabilities from the “evalSRE7.csv” file, the list of GO terms and relative genes from "AllGOprocesslabel.csv" and the list of all the genes linked to all the relative GO terms in from the "listGOterms_correct.txt" file. If this file does not exist the procedure creates it for the first time. The procedure applies the rules of the 3 consecutive probabilities above 0.9 to all the promoters and stores the resulting matrix to the “lethrmedia3prob09_full_stride10.csv" file. Then every GO groups is compared with all the remaining genes to have more genes with SRE-NFY couples detected. The enrichment in promoters with SRE-NRY couples is expressed as the Odds ratio with confidence intervals and a list of corresponding statistical p-values calculated by the “epitools::oddsratio.fisher” function. The results are stored in the “OddsRatiosGoterms_f64k3_prox3prob09_full_strid10.csv” file. Then, the GO groups are ranked for decreasing Odds ratio and the first 30 GO groups are analyzed in details for SRE-NFY occurrence. The results are stored in the ./GOgroups dir.

9_DeepSRE6_plot_prom_results

This routine collects all the data produced until now to create a graph for a chosen promoter, as in figure 6 for the LSS gene, in which is shown: 1) Individual probabilities of overlapping fragments as dots and red line, 2) smoothed probabilities as a gray line, 3) TFChIP ENCODE SRE peaks as blue bars, 4) TFChIP ENCODE NFY peaks as red bar, 5) green bars if that peak has been used to train the model, 6) SRE peaks from an independent source as blue shaded bar, 7) NFY peaks from an independent source as red shaded bar.

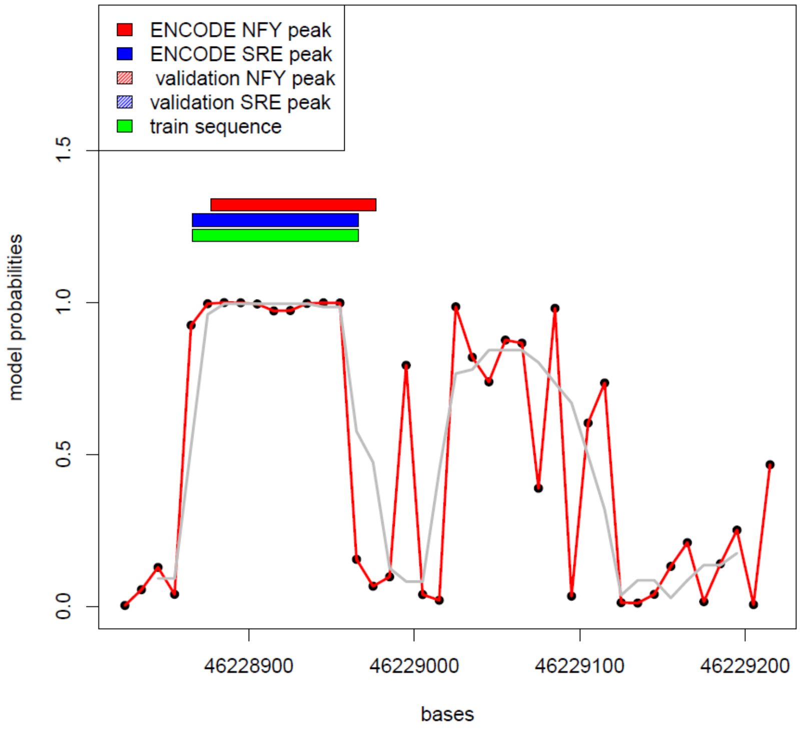

Step 10

The last part of the program is aimed to understand which bases are considered relevant to the model to predict the occurrence of a SRE-NFY couple in a promoter. To accomplish this task the procedure again chops the promoter in 100-bases overlapping fragments and perform the task on every single fragment. The procedure first predicts the base probability of a SRE-NFY couple for that fragment. Then the procedure “obscures” or “occludes” the first base by zeroing it and recalculates the probability. Then, it steps forward one base and repeats the calculation. At the end of the iterations, a list of 100 probabilities is obtained by occluding every consecutive base. A base is considered important for the model if its occlusion produces a relevant drop in model probability. Since fragments are overlapping, all the fragments plots are averaged position-wise and a final plot is produced for the whole 500 bp promoter.

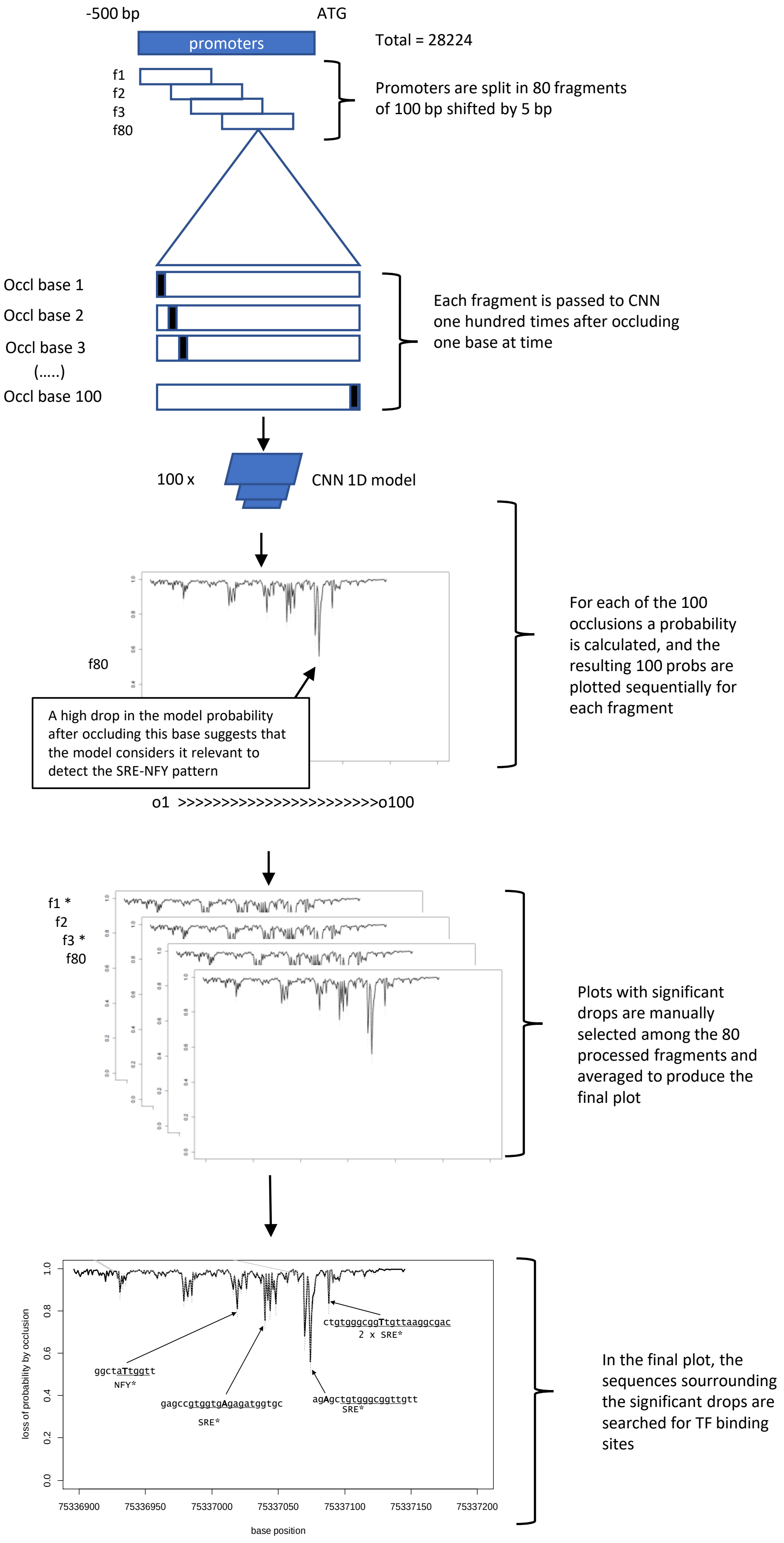

-500 bp
ATG
promoters
Total = 28224
f1
f2
f3
f80
Promoters are split in 80 fragments of 100 bp shifted by 5 bp
Occl base 1
Occl base 2
Occl base 3
(.....)
Occl base 100
Each fragment is passed to CNN one hundred times after occluding one base at time
100 x
CNN 1D model
f80
A high drop in the model probability after occluding this base suggests that the model considers it relevant to detect the SRE-NFY pattern
o1 >>>>>>>>>>>>>>>>>>>>>>>>o100
For each of the 100 occlusions a probability is calculated, and the resulting 100 probs are plotted sequentially for each fragment
f1 *
f2
f3 *
f80
Plots with significant drops are manually selected among the 80 processed fragments and averaged to produce the final plot
loss of probability by occlusion
1.0
0.8
0.6
0.4
0.2
0.0
ggctaTtggtt
NFY*
gagccgtggtgAgagatggtgc
SRE*
ctgtgggcggTtgttaaggcgac
2 x SRE*
agAgctgtgggcggttgtt
SRE*
75336900
75336950
75337000
75337050
75337100
75337150
75337200
base position
In the final plot, the sequences sourrounding the significant drops are searched for TF binding sites

10_DeepSRE6_promoter_occlusion

This procedure works for single promoters stores in the “Deep_promoters”directory

10b_DeepSRE6_ENCODEpeak_occlusion

This procedure works for training or control peaks stored in the ./posseq or ./negseq dirs.
